# Mapping antimicrobial resistance interventions in ASEAN countries: a scoping review of implementation patterns, One Health integration, and evidence gaps

**DOI:** 10.1186/s41182-026-00992-w

**Published:** 2026-05-30

**Authors:** Rahmatullah Nazari, Shabrina Arifia Qatrannada, Murtaza Jafari, Sayed Hussain Amiri, Christian Joseph N. Ong, Kevin Smith P. Cabuhat, Mohamed Mustaf Ahmed, Shuaibu Saidu Musa, Zhinya Kawa Othman, Olalekan John Okesanya, Zahroh Shaluhiyah, Don Lucero-Prisno Eliseo

**Affiliations:** 1 Medical Sciences Research Center, Asas Medical University, Kabul, Afghanistan; 2 Department of Health Promotion and Behavioral Sciences, Faculty of Public Health, Diponegoro University, Semarang, Indonesia; 3 Research Center, Shifa University, Kabul, Afghanistan; 4 Department of Paraclinic, Faculty of Curative Medicine, Shifa University, Kabul, Afghanistan; 5 Department of Microbiology, De La Salle University, Manila, Philippines; 6 Department of Biology, College of Science, De La Salle University, Manila, Philippines; 7 Faculty of Medicine and Health Sciences, SIMAD University, Mogadishu, Somalia; 8 School of Global Health, Faculty of Medicine, Chulalongkorn University, Bangkok, Thailand; 9 Faculty of Pharmaceutical Sciences, Chulalongkorn University, Bangkok, Thailand; 10 Department of Public Health and Maritime Transport, University of Thessaly, Volos, Greece; 11 Department of Global Health and Development, London School of Hygiene and Tropical Medicine, London, UK

**Keywords:** Antimicrobial resistance, One Health, Antimicrobial stewardship, Health systems, Southeast Asia, Scoping review

## Abstract

**Introduction:**

Antimicrobial resistance (AMR) is a critical and escalating public health threat globally, with Southeast Asia facing particularly high burdens due to elevated antimicrobial consumption, fragmented health systems, and uneven regulatory enforcement. Although member states of the Association of Southeast Asian Nations (ASEAN) have introduced AMR-related initiatives aligned with the Global Action Plan on AMR, the regional evidence base remains dispersed, fragmented across sectors, and insufficiently synthesized from a One Health perspective. This scoping review aimed to systematically map implemented AMR interventions across ASEAN countries, characterize their implementation patterns, assess the extent of One Health integration, and identify evidence gaps.

**Methods:**

We conducted a scoping review following PRISMA-ScR guidelines. PubMed/MEDLINE, Scopus, and ScienceDirect were searched for empirical studies published between January 1, 2018, and December 31, 2025, that reported the implementation of AMR-related programs, policies, or interventions in ASEAN member states. Data on country, sector, setting, intervention type, One Health pillar, study design, and outcomes were extracted and analyzed using narrative synthesis.

**Results:**

Of 9,832 records screened, 57 studies met the inclusion criteria. Evidence was heavily concentrated in three countries: Thailand (n = 15, 26.3%), Singapore (n = 13, 22.8%), and Vietnam (n = 12, 21.1%), accounting for 70.2% of all studies. Critically, no eligible peer-reviewed studies were identified from Brunei Darussalam, while Laos and Cambodia were represented only through disaggregated data from one multi-country surveillance study. The human health sector dominated (86.0%), while only 8.8% of studies adopted integrated One Health approaches, and no studies addressed the environmental sector in isolation. Antimicrobial stewardship was the most common intervention (63.2% of studies), but explicit use of implementation science frameworks was rare. Fifteen studies (26.3%) evaluated multi-component interventions, indicating an emerging shift toward integrated strategies. Most studies employed observational designs (50.9%) and assessed short-term process outcomes rather than sustained resistance reduction.

**Conclusions:**

This comprehensive mapping of AMR interventions across all ten ASEAN countries reveals extreme geographical and sectoral inequities. The complete absence of evidence from three member states and the dominance of hospital-based human health interventions underscore the urgent need for targeted investment in underrepresented settings and genuinely integrated One Health approaches. Without such advances, antimicrobial resistance initiatives in ASEAN will remain fragmented and unable to achieve sustained population-level impact.

**Supplementary Information:**

The online version contains supplementary material available at 10.1186/s41182-026-00992-w.

## Introduction

Antimicrobial resistance (AMR) represents one of the foremost global public health threats, directly responsible for an estimated 1.14 million deaths worldwide in 2021 [1], and projected to impose cumulative healthcare costs exceeding US$ 1 trillion by 2050 [2]. Although AMR is a natural evolutionary phenomenon, its acceleration is driven by interconnected human activities across health, agriculture, and environmental sectors, including irrational antibiotic prescribing, self-medication, and extensive antimicrobial use in livestock production [3–5].

In response to the growing AMR crisis, international organizations and regulatory authorities have introduced various policies and frameworks aimed at reducing antimicrobial misuse and strengthening surveillance. At the global level, the World Health Organization (WHO) endorsed the Global Action Plan on AMR in 2015, calling on member states to develop National Action Plans (NAPs) grounded in a One Health approach that integrates human, animal, and environmental health [6, 7].

AMR impacts nations across geographic regions, regardless of their economic status [8]. Across the globe, Asia consistently reports some of the highest resistance rates, underscoring the urgency of effective regional interventions [9]. Moreover, the burden of AMR is disproportionately high in Southeast Asia, defined in this review as the member states of the Association of Southeast Asian Nations (ASEAN), including Brunei, Cambodia, Indonesia, Laos, Malaysia, Myanmar, the Philippines, Singapore, Thailand, and Vietnam. During our study period, ASEAN comprised these 10 member states. Timor-Leste officially joined ASEAN on October 26, 2025 [10], near the end of our study period; therefore, it was not included as a focus of this review. The region has been identified as a global epicenter for emerging infectious diseases and AMR [6]. ASEAN countries face interrelated structural and contextual challenges that exacerbate AMR, including widespread antimicrobial use in agriculture, inadequate pharmaceutical regulations, limited laboratory capacity, weak surveillance systems, and socioeconomic barriers to healthcare access. Environmental drivers, including climate change, water contamination, and microplastic pollution, further facilitate bacterial proliferation and horizontal gene transfer. While most ASEAN countries have developed NAPs aligned with the WHO framework, the implementation of One Health strategies remains uneven, constrained by coordination gaps, limited resources, and insufficient evidence on intervention effectiveness [5, 11].

One Health offers a critical framework for addressing AMR because it recognizes that human, animal, and environmental health are inextricably linked [12]. Antimicrobial resistant bacteria and resistance genes do not respect sectoral boundaries. They circulate through humans, livestock, wildlife, food, water, and soil. Consequently, interventions confined to human healthcare settings alone cannot achieve sustained resistance reduction [13]. A One Health approach enables coordinated action across sectors, including optimizing antimicrobial use in both human and veterinary medicine, strengthening infection prevention in healthcare and agricultural settings, and monitoring environmental transmission pathways. Without such integration, AMR interventions risk addressing only part of the problem while leaving other drivers unchecked [14].

Several recent reviews have examined AMR in Southeast Asia, but they have important limitations that justify the need for a scoping review. While previous work has described One Health strategies in Southeast Asia [5], identified research priorities in Cambodia, Laos, and Vietnam [11], reported on NAP implementation progress across the WHO South-East Asia Region [15], and mapped regulatory frameworks across all ASEAN member states [16], None of these prior works quantified the geographical distribution of evidence, categorized interventions by sector and type, synthesized evaluation outcomes, or examined the use of theoretical frameworks. Consequently, a comprehensive, systematic synthesis of implemented AMR interventions across all ASEAN countries remains absent.

A scoping review methodology was selected rather than a systematic review for several reasons. First, our primary aim was to map the breadth, characteristics, and distribution of AMR interventions across ASEAN countries rather than to answer a focused clinical question or synthesize effect estimates. Second, scoping reviews are specifically designed to identify evidence gaps [17]and inform future research directions [18]. This is a core objective of the present study given the recognized fragmentation of AMR evidence in the region. Third, the heterogeneous nature of AMR interventions across human, animal, and environmental health sectors, coupled with the diversity of study designs and outcome measures, precludes meaningful quantitative synthesis, making a scoping review the most appropriate methodological choice. Finally, scoping reviews are well-suited for examining how research is conducted in a particular field [17], which aligns with our secondary aim of assessing evaluation quality, One Health integration, and the use of theoretical frameworks.

Accordingly, we conducted a scoping review to systematically map implemented AMR interventions across ASEAN countries. This review addresses this gap by providing the first comprehensive synthesis of implementation patterns, evaluation quality, and One Health integration across all 10 ASEAN member states.

## Methods

This scoping review was conducted following the Preferred Reporting Items for Systematic Reviews and Meta-Analyses extension for Scoping Reviews (PRISMA-ScR) [19]. The completed PRISMA-ScR checklist is provided in Supplementary File. The review protocol was not prospectively registered, as scoping reviews are currently ineligible for registration in PROSPERO under standard criteria. However, the review was conducted following a predefined protocol developed by the research team. The research question was structured using the PCC (Population, Concept, Context) framework appropriate for scoping reviews. The Population comprised ASEAN member states; the Concept encompassed implemented AMR interventions across any One Health sector; and the Context included any setting such as healthcare facilities, community, farms, or national level. Prior to formal screening, reviewers conducted a calibration exercise using a random sample of 50 records to align understanding of eligibility criteria. Title and abstract screening and full-text eligibility assessments were conducted independently by two reviewers (R.N. and S.A.Q.), and all discrepancies were resolved through discussion until consensus was reached. A third reviewer (M.J.) was available for adjudication when consensus could not be reached.

### Search strategy

A comprehensive literature search was conducted to identify empirical studies reporting the implementation of interventions to address AMR in ASEAN countries. PubMed/MEDLINE, Scopus, and ScienceDirect were selected for their comprehensive coverage of biomedical, public health, and interdisciplinary research relevant to AMR interventions. The search covered publications from 1 January 2018 to 31 December 2025. This timeframe was chosen to reflect the period during which NAP on AMR and One Health-oriented strategies have been increasingly implemented across ASEAN member states, following the May 2017 deadline established by the WHO Global Action Plan on AMR for member states to finalize such plans [7].

The search strategy was developed through an iterative process involving initial scoping searches and refinement based on the yield of known relevant studies. To maximize sensitivity, we combined controlled vocabulary (Medical Subject Headings [MeSH] where available) with an extensive range of free-text synonyms for each conceptual domain. The AMR concept included terms such as 'antimicrobial resistance,' 'antibiotic resistance,' 'drug resistance,' 'AMR,' 'MDR,' 'ESBL,' 'MRSA,' and 'carbapenem resistant.' The intervention concept encompassed 'intervention,' 'program,' 'policy,' 'strategy,' 'stewardship,' 'surveillance,' 'infection prevention and control,' 'IPC,' 'education,' and 'behavior change.' Search terms also encompassed antimicrobial use concepts including 'antibiotic use,' 'antimicrobial consumption,' and 'antibiotic prescribing' to capture interventions aimed at reducing AMU as a pathway to mitigating AMR. The geographical concept included all ten ASEAN country names along with alternative designations including 'Burma' for Myanmar, 'Lao PDR' for Laos, and 'Brunei Darussalam' for Brunei. Boolean operators (AND/OR) were used to link concepts while maintaining conceptual coherence. The strategy was piloted against a test set of known eligible studies to verify capture. The full search strings for all databases (PubMed, Scopus, and ScienceDirect), including search dates, filters, and record counts, are provided in the Supplementary File (Part B).

### Eligibility criteria

Studies that evaluated a formal initiative (e.g., a program or policy) designed to combat AMR were included. The study's background section was required to explicitly frame the intervention within the context of AMR, even if reducing resistance was not its primary outcome of interest [20]. Interventions targeting human, animal, or environmental health were eligible, with no restrictions on scale (local, national, or regional). Studies conducted in clinical, community, institutional, and policy settings were considered, encompassing a range of study designs, including observational, quasi-experimental, experimental, human, and veterinary research.

To be included, studies had to provide a clear description of the intervention components or activities implemented and explicitly specify the country or region of implementation within Southeast Asia. Given our focus on the ASEAN, we required that studies be conducted entirely within one or more ASEAN member states or present disaggregated data that allowed the extraction of findings specific to ASEAN countries. Studies that included both ASEAN and non-ASEAN countries were included only if they presented disaggregated data allowing extraction of findings specific to ASEAN countries. Studies without separable data were excluded.

We excluded: (i) non-empirical publications (systematic reviews, bibliometric analyses, and narrative reviews); (ii) study protocols; (iii) studies focusing exclusively on viral, parasitic, or fungal resistance; (iv) purely descriptive studies without intervention implementation; and (v) studies published in languages other than English. The review was restricted to English-language publications to ensure consistency in interpretation and data extraction; although this may introduce language bias, it was necessary for methodological rigor. The review was restricted to peer-reviewed empirical studies indexed in PubMed, Scopus, and ScienceDirect. Grey literature, including government reports, non-governmental organization publications, policy briefs, and multilateral agency documents, was not systematically searched, which we acknowledge as a limitation.

### Selection process

The study selection process was conducted in accordance with the Preferred Reporting Items for Systematic Reviews and Meta-Analyses Extension for Scoping Reviews guidelines. All retrieved records were imported into Mendeley Reference Manager for deduplication and data management. Duplicate records were identified using the software's built-in 'Check for Duplicates' feature, which compares bibliographic fields such as title, author names, publication year, and source. All flagged duplicates were manually verified before removal to ensure accuracy. After removing 5,208 duplicate records, 4,624 unique records remained for title and abstract screening. The software was also used to organize and track records throughout the title/abstract and full-text screening process. Reviewers (R.N. and S.A.Q.) were members of the research team with familiarity with the AMR literature in ASEAN and received training in the systematic review methodology employed in this study. The third reviewer (M.J.) was selected based on senior research experience to serve as adjudicator when consensus could not be reached. Prior to formal screening, reviewers (R.N. and S.A.Q.) conducted a calibration exercise using a random sample of 50 records to align understanding of eligibility criteria. Title and abstract screening and full-text eligibility assessments were then conducted independently by the two reviewers in a non-blinded manner, as reviewer familiarity with the literature was considered beneficial for accurate eligibility assessment, and all discrepancies were resolved through discussion until consensus was reached. A third reviewer (M.J.) was available for adjudication when consensus could not be reached.

## Quality appraisal

Consistent with established guidance for scoping reviews [19], formal risk of bias assessment or critical appraisal of individual studies was not conducted. Scoping reviews aim to map the breadth and characteristics of available evidence rather than to synthesize effect estimates or determine intervention effectiveness. Accordingly, all studies meeting the inclusion criteria were included regardless of methodological quality. This approach aligns with PRISMA-ScR guidelines, which do not mandate quality appraisal for scoping reviews.

### Data extraction

Data were extracted from the included studies using a pre-designed data extraction form (Supplementary File, Part C) that was piloted on five randomly selected studies and refined accordingly. Data extraction was independently performed by two reviewers (R.N. and S.A.Q.), and disagreements were resolved through discussion or consultation with a third reviewer (M.J.).

The extracted variables included authors, year of publication, title, country, source type, sector (human, animal, or environmental health, or multisectoral/One Health), study setting (healthcare facility, community, animal farm, multi-setting, or national level), intervention category, specific intervention activities, One Health pillar addressed (intervention, surveillance, policy and economics, behavioral insights and change, or multi-pillar), and study design. In addition, we recorded whether an evaluation was conducted, the primary outcomes measured, and key findings reported in each study.

All included studies were evaluative in nature and reported the outcomes of implemented AMR interventions. Subsequently, a narrative synthesis was conducted to describe the characteristics, scope, and distribution of AMR-related interventions across sectors and settings, and to identify gaps in intervention evaluation and implementation.

## Results

### Study selection

Following a systematic database search, 9,832 records were identified: PubMed (n = 749), ScienceDirect (n = 5,805), and Scopus (n = 3,278). Duplicate records (n = 5,208) were identified and removed before screening, resulting in 4,624 unique records for further assessment.

In the initial title and abstract screening, 4,510 records were excluded because they were clearly irrelevant to the research question, leaving 114 records for full-text retrieval. Of these, a total of 25 records were excluded due to full-text unavailability after database and institutional access screening. Metadata screening indicated that these studies were primarily conducted in Vietnam, Indonesia, and Cambodia. No additional retrieval strategies (e.g., interlibrary loan or author contact) were pursued, as this was beyond the predefined review protocol.

**Fig. 1:**
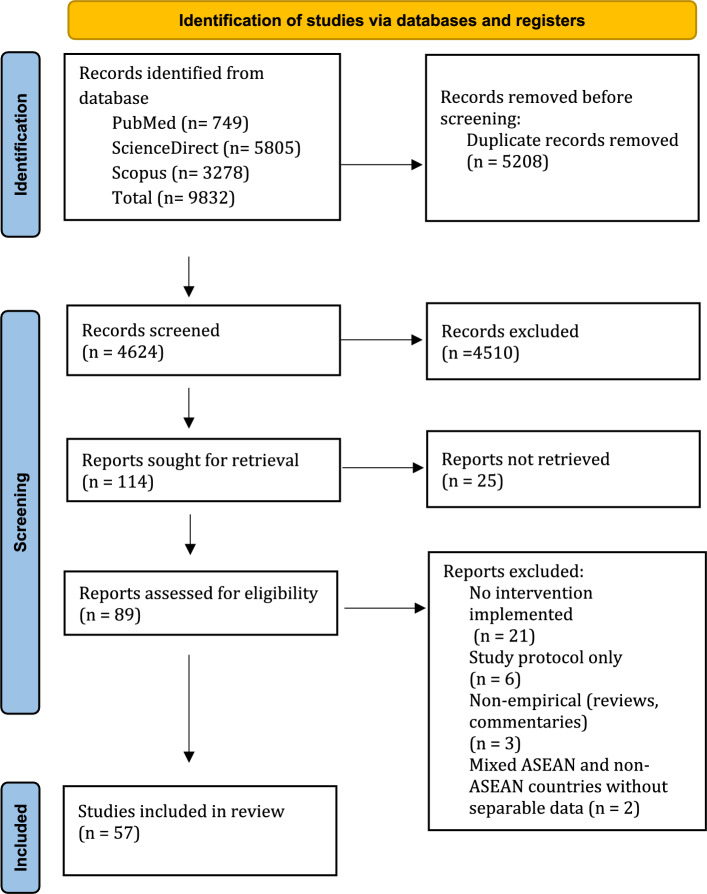
PRISMA chart showing process of the included and excluded reports

At the full-text assessment stage, 32 articles were excluded for reasons, including no intervention implemented, study protocols, non-empirical publications, and mixed ASEAN/non-ASEAN publications without separable data (Fig. 1). Ultimately, 57 studies fulfilled all eligibility criteria and were included in the final synthesis.

### Characteristics of included studies

A total of 57 studies met the inclusion criteria. Publication years ranged from 2018 to 2025, with output peaking in 2020 (n = 13) and 2021 (n = 10). This peak likely reflects the maturation of the National Action Plans implemented after 2017. A decline in 2022–2023 (n = 6 and n = 3) may be attributable to the COVID-19 pandemic diverting research resources, followed by a rebound in 2025 (n = 10).

**Fig. 2:**
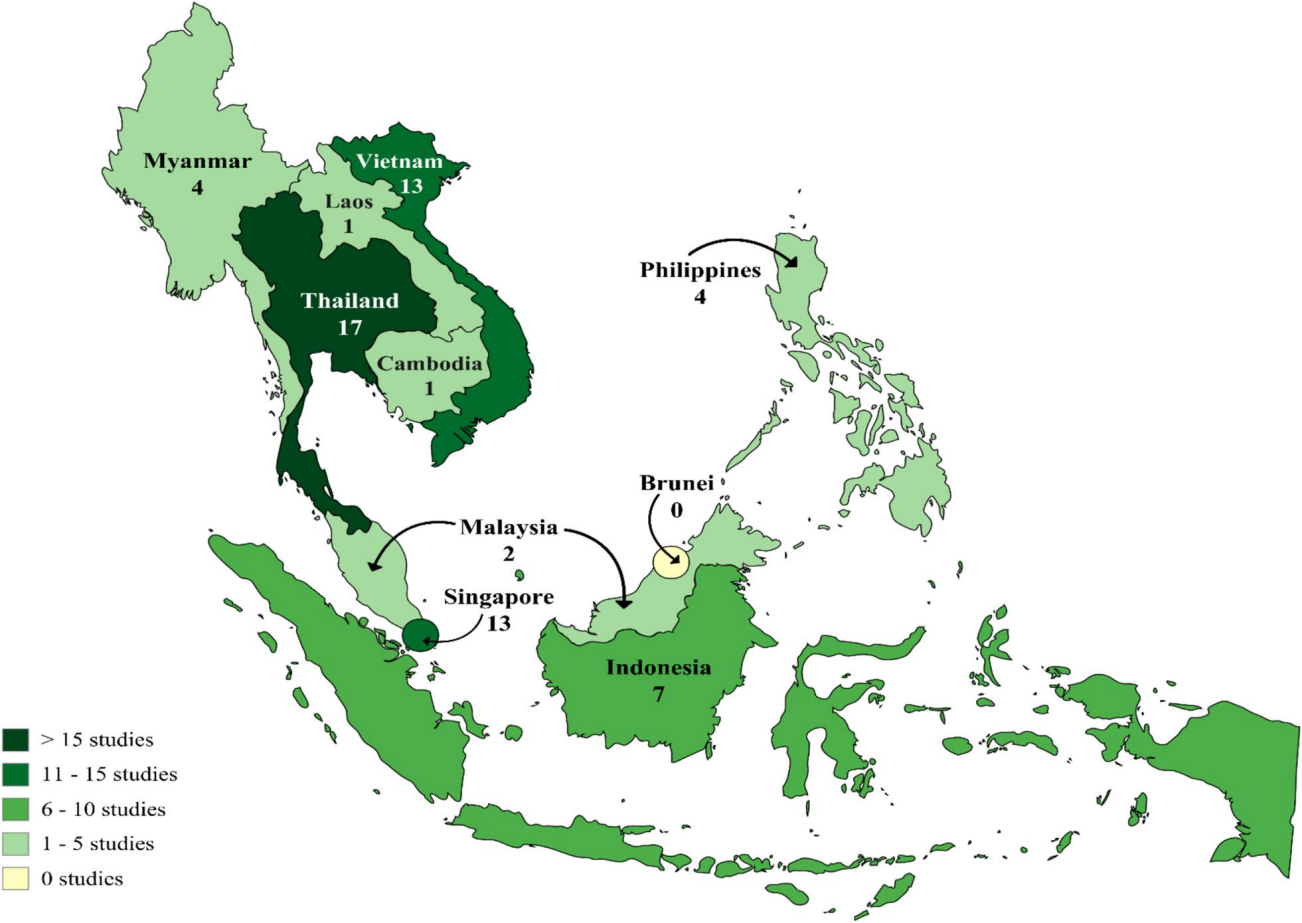
Geographical distribution of AMR intervention studies across ASEAN member states. Studies conducted in multiple countries are counted for each participating country; therefore, the sum of country counts (n = 62) exceeds the total number of included studies (n = 57). Thailand (n = 17), Singapore (n = 13), and Vietnam (n = 13) accounted for the majority of studies. Indonesia contributed seven studies, the Philippines four, Myanmar four, Malaysia two, and Cambodia and Laos one each (both from a single multi-country surveillance initiative. No eligible peer-reviewed studies were identified from Brunei Darussalam

The geographical distribution of the included studies was markedly uneven. As shown in Fig. 2, most of the evidence originated from three countries: Thailand (n = 15, 26.3%), Singapore (n = 13, 22.8%), and Vietnam (n = 12, 21.1%), which together accounted for 40 studies (70.2%). Indonesia contributed seven studies (12.3%), followed by the Philippines (n = 4, 7.0%). Malaysia and Myanmar each contributed two studies (3.5% each). One multi-country study included five ASEAN countries (Cambodia, Laos, Myanmar, Thailand, and Vietnam) and is counted once (1.8%). Another study (1.8%) was conducted across Thailand and Myanmar. No eligible peer-reviewed studies were identified from Brunei Darussalam in this review. The limited availability of peer-reviewed evaluative evidence from Brunei Darussalam represents a critical gap in the indexed literature that hinders comprehensive understanding of the regional AMR intervention landscape.

**Fig. 3:**
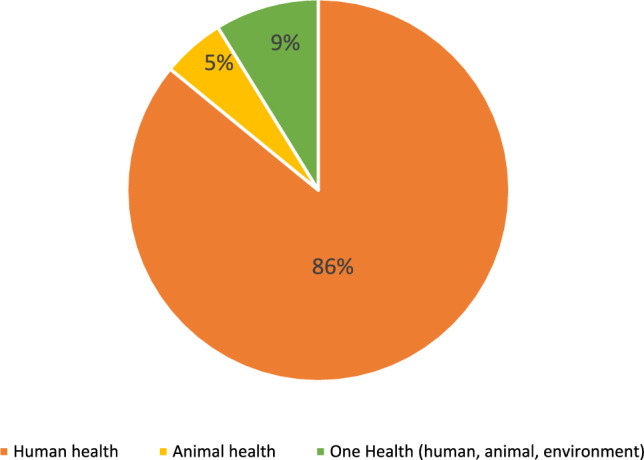
Sectoral distribution of AMR intervention studies in ASEAN (n = 57). Human health studies dominated the evidence base (86%), while only 9% of studies adopted a One Health approach encompassing human, animal, and environmental health. No studies addressed the environmental sector in isolation

By sector, the included studies were heavily concentrated in the human health sector, accounting for 49 studies (86.0%). Only three studies (5.3%) focused exclusively on animal health, and no studies addressed the environmental sector in isolation. Critically, only five studies (8.8%) adopted a multisectoral One Health approach encompassing human, animal, and environmental health simultaneously. This sectoral imbalance highlights a significant gap in the evidence base, particularly the lack of integrated approaches essential for addressing the complex drivers of AMR (Fig. 3).

Consistent with the sectoral concentration in human health, the included studies were predominantly conducted in healthcare facilities, accounting for 47 studies (82.5%). Community-based settings were represented in two studies (3.5%), while one study each was conducted on animal farms (1.8%) and in veterinary clinics (1.8%). Three studies (5.4%) were multi-setting, and one study was implemented at the national level (1.8%). This distribution reinforces the focus on healthcare facilities observed in the sectoral analysis, with limited representation of community, animal, and integrated settings.

When categorized according to the One Health framework, the majority of studies aligned primarily with the intervention pillar, accounting for 31 studies (54.4%). Surveillance was the next most frequently addressed pillar, with nine studies (15.8%) focused on surveillance activities. Behavioral insights and change was the primary focus of six studies (10.5%), while policy and economics alone was addressed in two studies (3.5%). An additional nine studies (15.8%) adopted a multi-pillar approach, integrating two or more One Health components. The emergence of multi-pillar approaches, though still limited, indicates growing recognition of the need for integrated strategies that simultaneously address behavioral, policy, and surveillance dimensions of AMR.

In addition to the distribution across One Health pillars, the included studies varied considerably in their research designs. Observational studies predominated, accounting for 29 studies (50.9%). Quasi-experimental designs were used in 13 studies (22.8%), and experimental designs in six studies (10.5%). Mixed-methods approaches were employed in four studies (7.0%), qualitative studies in three (5.3%), and one mathematical modeling study (1.8%). One study (1.8%) incorporated both quasi-experimental and observational elements. The dominance of observational designs reflects real-world implementation challenges, whereas the limited number of experimental and mixed-methods studies highlights opportunities for strengthening the evidence base through more rigorous and integrated approaches.

The detailed distributions of the publication year, country, setting, One Health pillar, and study design are presented in Table 1.

**Table 1:** Characteristics of Included Studies (n = 57)

Characteristic	Number of studies	Percentage (%)
Publication Year		
2018	5	8.8
2019	6	10.5
2020	13	22.8
2021	10	17.5
2022	6	10.5
2023	3	5.3
2024	4	7.0
2025	10	17.5
Country		
Thailand	15	26.3
Singapore	13	22.8
Vietnam	12	21.1
Indonesia	7	12.3
Philippines	4	7.0
Malaysia	2	3.5
Myanmar	2	3.5
Cambodia, Laos, Myanmar, Thailand, Vietnam	1	1.8
Thailand & Myanmar	1	1.8
Setting		
Healthcare Facility	47	82.5
Multi-setting	3	5.3
Community	2	3.5
Animal Farms	1	1.8
Healthcare Facility/Community	1	1.8
Veterinary clinics	1	1.8
National Level	1	1.8
Not Specified	1	1.8
One Health Pillar		
Intervention	31	54.4
Surveillance	9	15.8
Behavioral Insights & Change	6	10.5
Policy & Economics	2	3.5
Multi-pillar approaches	9	15.8
Study Design		
Observational	29	50.9
Quasi-experimental	13	22.8
Experimental	6	10.5
Mixed-methods	4	7.0
Qualitative study	3	5.3
Quasi-experimental /observational	1	1.8
Mathematical modeling	1	1.8

### Intervention type and distribution

A total of 57 antimicrobial resistance (AMR) interventions were identified and categorized according to their primary implementation focus. Antimicrobial stewardship was the most frequently employed strategy, 27 studies (47.4%) evaluating standalone stewardship programs. Nine additional studies (15.8%) combined antimicrobial stewardship with other approaches, including awareness, capacity building, training, program adaptation, surveillance, and policy components. A total of 36 studies (63.2%) incorporated antimicrobial stewardship as a component, underscoring its role in regional AMR containment strategies.

Surveillance-focused interventions were the second most common category, with nine studies (15.8%) evaluating surveillance as the primary intervention and six additional studies (10.5%) integrating surveillance with other strategies, such as policy – regulation, infection prevention and control, awareness, and antimicrobial stewardship. Awareness-based interventions accounted for four studies (7.0%) as standalone approaches, with an additional seven studies (12.3%) combining awareness with other approaches. Capacity building alone was assessed in one study (1.8%) and featured in combination with other strategies in seven additional studies (12.3%). IPC was the primary focus of one study (1.8%) and featured in two multi-component interventions (3.5%). Similarly, policy and regulatory interventions appeared alone in one study (1.8%) and in combination with other interventions in seven studies (12.3%), most often combined with stewardship and surveillance components, reflecting the growing recognition that regulatory frameworks need to be integrated with implementation strategies.

**Fig. 4:**
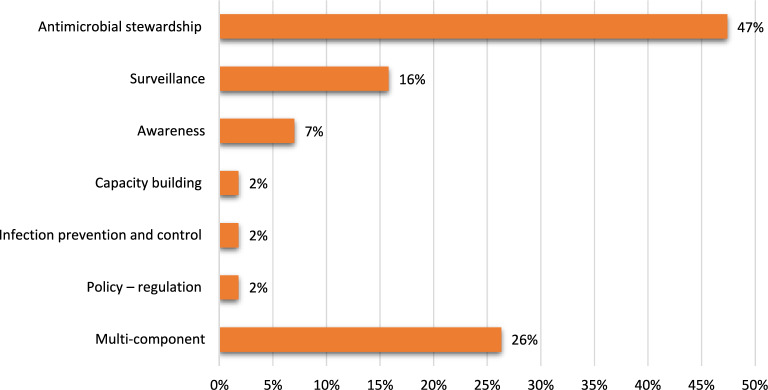
Types of AMR interventions evaluated in ASEAN (n = 57). Categories are not mutually exclusive; 15 studies (26%) evaluated multi-component interventions combining two or more strategies. Percentages therefore sum to more than 100%. Values are rounded to the nearest integer

Overall, 15 studies (26.3%) evaluated multi-component interventions combining two or more strategies, indicating an emerging shift toward integrated approaches to AMR (Fig. 4).

### Summary of intervention outcomes

Tables 2 and 3 present the detailed characteristics and key findings of the 57 evaluated AMR interventions across ASEAN countries. Table 2 provides contextual information for each study, including country, sector, setting, One Health pillar, and study design. Table 3 summarizes the intervention type, description of the intervention activity, primary outcome measured, and key findings for each study. Studies in both tables are organized by intervention type to facilitate comparison. A synthesis of the patterns emerging from these findings is presented in the Discussion.

**Table 2:** Description of evaluation studies (n = 57)

First author	Publication year	Country	Sector	Setting	One Health Pillars	Study design
Chia et al. [21]	2019	Singapore	Human health	Healthcare facility	Intervention	Observational
Loo et al. [22]	2019	Singapore	Human health	Healthcare facility	Intervention	Observational
Ng et al. [23]	2023	Singapore	Human health	Healthcare facility	Intervention	Observational
Wee et al. [24]	2020	Singapore	Human health	Healthcare facility	Intervention	Observational
Wong et al. [25]	2020	Singapore	Human health	Healthcare facility	Behavioral Insights and Change	Experimental
Ginting et al. [26]	2019	Indonesia	Human health	Healthcare facility	Surveillance	Observational
Nguyen et al. [27]	2022	Vietnam	Human health	Healthcare facility	Intervention	Observational
Sani et al. [28]	2025	Indonesia	Animal health	Animal Farms	Intervention	Quasi-experimental
Haenssgen et al. [29]	2018	Thailand	Human health	Healthcare facility	Intervention	Observational
Doshi et al. [30]	2025	Vietnam	Human health	Healthcare facility	Intervention	Quasi-experimental
Sirijatuphat et al. [31]	2020	Thailand	Human health	Healthcare facility	Surveillance	Observational
Betito et al. [32]	2021	Philippines	Human health	Healthcare facility	Intervention	Observational
Huong et al. [33]	2021	Vietnam	Human health	Healthcare facility	Intervention	Mixed-methods
Booton et al. [34]	2021	Thailand	Human health, Animal health, Environment	Multi-setting	Intervention /Transmission /Policy and Economics)	Mathematical modeling study (compartmental ODE model)
Natadidjaja et al. [35]	2024	Indonesia	Human health	Healthcare facility	Intervention	Observational
Herawati et al. [36]	2020	Indonesia	Human health	Healthcare facility	Intervention	Observational
Doshi et al. [37]	2025	Vietnam	Human health	Healthcare facility	Intervention	Experimental
Huang et al. [38]	2024	Singapore	Human health	Healthcare facility	Behavioral Insights and Change	Experimental
Valenzuela et al. [39]	2025	Philippines	Human health, Animal health, Environment	Multi-setting	Policy and Economics	Mixed-methods
Sinto et al. [40]	2024	Indonesia	Human health	Healthcare facility	Surveillance	Mixed-methods
Loo et al. [41]	2020	Singapore	Human health	Healthcare facility	Intervention	Observational
Guo et al. [42]	2025	Singapore	Animal health	Veterinary clinics	Behavioral Insights and Change	Qualitative study
Nguyen-Thi et al. [43]	2023	Vietnam	Human health	Healthcare facility	Intervention	Quasi-experimental
Jitmuang et al. [44]	2020	Thailand	Human health	Healthcare facility	Surveillance	Observational
Qibtiyah et al. [45]	2023	Indonesia	Human health	Healthcare facility	Intervention	Observational
Jantarathaneewat et al. [46]	2021	Thailand	Human health	Healthcare facility	Intervention	Quasi-experimental
Sirijatuphat et al. [47]	2022	Thailand	Human health	Healthcare facility	Surveillance	Quasi-experimental
Wangchinda et al. [48]	2022	Thailand	Human health	Healthcare facility	Intervention	Quasi-experimental
Thomnoi et al. [49]	2022	Thailand	Human health	Healthcare facility	Intervention	Quasi-experimental
Loo et al. [50]	2025	Singapore	Human health	Healthcare facility	Behavioral Insights and Change /Intervention	Observational
Aoybamroong et al. [51]	2019	Thailand	Human health	Healthcare facility	Behavioral Insights and Change /Intervention	Quasi-experimental
Chautrakarn et al. [52]	2019	Thailand	Human health	Healthcare facility	Intervention	Observational
Huong et al. [53]	2021	Vietnam	Human health	Healthcare facility	Intervention	Mixed-methods
Waleekhachonloet et al. [54]	2021	Thailand	Human health	Healthcare facility	Policy and Economics /Intervention /Surveillance /Behavioral Insights and Change	Quasi-experimental
Arulappen et al. [55]	2021	Malaysia	Human health	Healthcare facility	Intervention	Observational
Wai et al. [56]	2018	Myanmar	Human health	Community	Intervention	Observational
Althaus et al. [57]	2019	Thailand, Myanmar	Human health	Healthcare facility	Intervention	Experimental
Thamlikitkul et al. [58]	2020	Thailand	Human health	Healthcare facility	Surveillance	Observational
Karyanti et al. [59]	2020	Indonesia	Human health	Healthcare facility	Intervention	Quasi-experimental
Nguyen-Thi et al. [60]	2021	Vietnam	Human health	Healthcare facility	Intervention	Observational
Thong et al. [61]	2021	Malaysia	Human health	Healthcare facility	Behavioral Insights and Change	Experimental
Phan et al. [62]	2022	Vietnam	Human health	Healthcare facility	Intervention	Quasi-experimental
Yong et al. [63]	2025	Singapore	Human health	Healthcare facility	Intervention	Quasi-experimental
Woo et al. [64]	2018	Singapore	Human health	Healthcare facility	Intervention	Quasi-experimental
Liew et al. [65]	2020	Singapore	Human health	Healthcare facility	Intervention	Observational
Bâtie et al. [66]	2025	Vietnam	Animal health	Multi-setting	Policy and Economics/Behavioral Insights and Change	Observational
Lota et al. [67]	2022	Philippines	Human health, Animal health, Environment	Not specified	Policy and Economics	Observational
Sumpradit et al. [68]	2021	Thailand	Human health, Animal health, Environment	Multi-setting	Policy and Economics/Surveillance/Intervention/Behavioral Insights and Change	Observational
Bordier et al. [69]	2018	Vietnam	Human health, Animal health, Environment	National level	Multi-pillar	Observational
Sirijatuphat et al. [70]	2018	Thailand	Human health	Healthcare facility	Surveillance	Observational
Drabarek D., et al. [71]	2025	Vietnam	Human health	Healthcare facility/Community	Intervention	Observational
Swe et al. [72]	2020	Myanmar	Human health	Community	Behavioral Insights and Change	Observational
Phan et al. [73]	2020	Vietnam	Human health	Healthcare facility	Infection Prevention and Control/Behavioral Insights and Change	Quasi-experimental/Observational
Argimón et al. [74]	2020	Philippines	Human health	Healthcare facility	Surveillance	Observational
Anugulruengkitt et al. [75]	2025	Thailand	Human health	Healthcare facility	Behavioral Insights and Change	Qualitative study
Cheng et al. [76]	2024	Singapore	Human health	Healthcare facility	Intervention	Experimental
Lim et al. [77]	2020	Cambodia, Laos, Myanmar, Thailand, Vietnam	Human health	Healthcare facility	Surveillance	Observational

**Table 3:** Characteristics and key findings of evaluated AMR intervention studies in ASEAN countries (n = 57)

Author (Year)	Title of study	Type of intervention	Intervention Activity (Short)	Primary Outcome Measured	Key Finding
Chia et al. [21]	Do antimicrobial stewardship programme interventions reduce the rate of and protect against Clostridium difficile infection?	Antimicrobial stewardship	AMS program with audit & feedback	Healthcare-associated CDI rates	No reduction in primary CDI incidence and mortality; lower recurrence and shorter therapy when ASP is accepted
Loo et al. [22]	Discontinuation of antibiotic therapy within 24 h of treatment initiation for patients with no clinical evidence of bacterial infection: a 5-year safety and outcome study from Singapore General Hospital Antimicrobial Stewardship Program	Antimicrobial stewardship	Daily audit + early discontinuation recommendation	Duration of therapy, LOS, cost	Shorter antibiotic duration (2.72 vs. 5.33 days) and LOS; saved ~ SGD 10,817/patient
Ng et al. [23]	Antibiotic utilisation and resistance over the first decade of nationally funded antimicrobial stewardship programmes in Singapore acute care hospitals	Antimicrobial stewardship	National ASP funding, guidelines, education, CDSS	Broad-spectrum antibiotic use & resistance	Decreased broad-spectrum use and resistant organisms over a decade
Wee et al. [24]	Who listens and who doesn’t? Factors associated with adherence to antibiotic stewardship intervention in a Singaporean tertiary hospital	Antimicrobial stewardship	Prospective audit & feedback by ASP team	Adherence to ASP interventions	81.9% adherence; phone calls improved compliance
Wong et al. (2020) [25]	Empowerment of nurses in antibiotic stewardship: a social ecological qualitative analysis	Awareness	Nurse FGDs on ASP involvement (Social Ecological Model)	Nurse perceptions, barriers, facilitators	Nurses feel empowered but lack AMR knowledge and need formal training and role recognition
Ginting et al. [26]	Rethinking Antimicrobial Resistance Surveillance: A Role for Lot Quality Assurance Sampling	Surveillance	Implemented LQAS methodology for AMR surveillance in 11 Indonesian sites; evaluated diagnostic accuracy, cost, and time to results	Sensitivity, specificity, time to results, cost	LQAS had > 98% sensitivity for detecting high-resistance populations; cost US$466 per site; time to results was 47–138 days; and detected local variations in AMR prevalence
Nguyen et al. (2022) [27]	Effectiveness of an enhanced antibiotic stewardship programme among paediatric patients in a tertiary hospital in Vietnam	Antimicrobial stewardship	Enhanced ASP with pre-authorization & guidelines	Antibiotic consumption & AWaRe prescribing	Improved Access antibiotic use and Access-to-Watch ratio, but had limited impact on overall antibiotic consumption with no change in clinical outcomes
Sani et al. [28]	Longitudinal evaluation of interventions on antimicrobial use and antimicrobial resistance on broiler farms in West Java, Indonesia	Antimicrobial stewardship/Awareness/Training	Multi-component farm intervention (training, benchmarking)	Antimicrobial use & resistance in E. coli	Colistin use decreased by 92.6% (due to a national ban); no reduction in AMR
Haenssgen et al. [29]	The social role of C-reactive protein point-of-care testing to guide antibiotic prescription in Northern Thailand	Antimicrobial stewardship	CRP POCT + patient education video	Antibiotic prescription rates	CRP testing reduces antibiotic prescribing and has high patient acceptance
Doshi et al. [30]	Feasibility of an antimicrobial stewardship program in four district hospitals in Vietnam	Antimicrobial stewardship	AMS committees, guidelines, training, audit/feedback	Antibiotic consumption & prescribing appropriateness	Modest reductions in antibiotic use and mortality; inappropriate prescriptions remained high (~ 80%)
Sirijatuphat et al. [31]	Implementation of global antimicrobial resistance surveillance system (GLASS) in patients with bacteriuria	Surveillance	GLASS implementation for bacteriuria	Infection vs colonization, AMR patterns	GLASS is feasible and more informative than standard laboratory-based surveillance. HAIs predominated, and were associated with more resistant pathogens compared to CAIs
Betito et al. [32]	Implementation of a multidisciplinary antimicrobial stewardship programme in a Philippine tertiary care hospital: an evaluation by repeated point prevalence surveys	Antimicrobial stewardship	Multidisciplinary ASP (education, restriction)	Prescribing quality, surgical prophylaxis	Improved documentation, reduced prolonged prophylaxis, and high Watch antibiotic use persisted
Huong et al. [33]	Improving antimicrobial use through antimicrobial stewardship in a lower-middle income setting: a mixed-methods study in a network of acute-care hospitals in Viet Nam	Antimicrobial stewardship	Mixed-methods assessment of AMS implementation	Staff perceptions, AMS core elements	Implementation varied by leadership and resources. Active programs showed stronger controls and audits. Staff supported AMS. Barriers: understaffed and undertrained roles, poor lab trust, and weak IT. AMR perceptions often inaccurate (esp. surgery). Need: staffing standards, training, IT, and collaboration
Booton et al. [34]	One Health drivers of antibacterial resistance: Quantifying the relative impacts of human, animal and environmental use and transmission	Policy—Regulations/Antimicrobial Stewardship/Surveillance	One Health mathematical modeling of ABR transmission	Human colonization with ESBL-E. coli	Human antibiotic use is the most impactful driver in lowering AMR; NSP-AMR could reduce colonization by 6%–18.8% over 20 years
Natadidjaja et al. [35]	A survey on define daily dose of watch- and access-category antibiotics in two Indonesian hospitals following the implementation of digital antimicrobial stewardship tool	Antimicrobial stewardship	Digital AMS tool (e-RASPRO) with AWaRe integration	DDD of Watch/Access antibiotics	In Hospital 1, implementation was associated with a 49% reduction in Watch-category antibiotics In Hospital 2, antibiotic use showed mixed trends, with an initial increase in Watch-category antibiotics, alongside decreases in some classes and a slight rise in Access-category use
Herawati et al. [36]	Interview-based cross-sectional needs assessment to advance the implementation of an effective antibiotic stewardship program in Indonesian hospitals	Antimicrobial Stewardship/Capacity Building/Surveillance/Policy—Regulations	Hospital readiness assessment via structured interviews	ASP readiness scores (0–10)	Readiness varied; public hospitals scored higher; IT and guideline gaps were identified
Doshi et al. [37]	The effect of antimicrobial stewardship interventions upon antimicrobial consumption and appropriateness in Vietnamese district hospitals: a cluster randomised trial	Antimicrobial stewardship	Cluster RCT of multifaceted AMS in district hospitals	Inappropriate prescribing, consumption	Inappropriate prescribing decreased by 6.3%, while total antimicrobial consumption remained unchanged
Huang et al. [38]	An Evidence-Based Serious Game App for Public Education on Antibiotic Use and Resistance: Randomized Controlled Trial	Awareness	Serious game app for public education on AMR	Knowledge, attitude, perception (KAP) scores	App improved KAP scores; 95% agreed that it raised awareness
Valenzuela et al. [39]	Situational analysis of antimicrobial resistance policies and program implementation in the Philippines, 2019–2023	policy—regulations/Surveillance/Capacity building/Awareness/Antimicrobial stewardship	Mixed-methods review of Philippine National Action Plan	PNAP progress, strengths, gaps	Progress in policy, regulation, and surveillance in human and animal health sectors, but environmental engagement remains weak, with gaps in lab capacity, data systems, and local coordination
Sinto et al. [40]	A nationwide mixed-methods study of gaps and barriers to implementation of antimicrobial stewardship programmes in hospitals in Indonesia	Surveillance	Nationwide mixed-methods evaluation of ASP implementation in 575 Indonesian hospitals using surveys and focus groups	ASP development scores (0–100%) across 6 domains; barriers and enablers	Most hospitals had ASPs (89.7%), but implementation was moderate and uneven, with strengths in leadership and training but gaps in infrastructure and monitoring, influenced by hospital capacity and regional development
Guo et al. [42]	The VALUE of antibiotic stewardship for companion animals: Understanding appropriate antibiotic prescribing for pet cats and dogs in veterinary clinics in Singapore	Awareness/Capacity Building	Qualitative study exploring antibiotic prescribing practices of 19 veterinarians using the VALUE model	Themes related to antibiotic prescribing practices, barriers, and enablers	Business viability prioritized; stewardship driven by individual values. Barriers: unregulated sales, fear of losing clients. Facilitators: peer networks, ethics, trust
Loo et al. [41]	Discontinuation of Antibiotics in Patients with Neurological Conditions –A Study on the Impact of an Antimicrobial Stewardship Program (ASP) in a Tertiary Institution	Antimicrobial stewardship	ASP for neurology patients (audit + PCT guidance)	Antibiotic duration, LOS, mortality	Shorter PLOS duration by 2 days and shorter antibiotic duration (4.99 vs. 6.66 days) with no safety compromise
Nguyen-Thi et al. [43]	Evaluation of the impact before and after the application of an antimicrobial stewardship program at Dong Thap General Hospital, Vietnam, from 2017 to 2021	Antimicrobial stewardship	ASP enhancement with training & monitoring	Antibiotic consumption (DOT/LOT/DDD)	DOT/LOT decreased post-ASP; DDD remained unchanged; and Watch antibiotics remained dominant
Jitmuang et al. [44]	Implementation of the World Health Organization’s Global Antimicrobial Resistance Surveillance System (GLASS) for the Surveillance of Sputum Specimens Collected from Patients at Siriraj Hospital	Surveillance	GLASS for sputum specimens	Infection vs colonization, AMR, outcomes	GLASS was feasible and provided more comprehensive AMR surveillance data, highlighting higher resistance, cost and worse outcomes in HAI
Qibtiyah et al. [45]	The impact of antimicrobial stewardship on reserve antibiotic use and procuring cost	Antimicrobial stewardship	Pre-authorization for meropenem (reserve antibiotic)	Meropenem consumption & cost	Meropenem consumption reduced by 53% and procurement costs dropped by 79%
Jantarathaneewat et al. [46]	Pharmacist-Driven Antibiotic Stewardship Program in Febrile Neutropenic Patients: A Single Site Prospective Study in Thailand	Antimicrobial stewardship	Pharmacist-driven ASP for febrile neutropenia	Antibiotic appropriateness, dosage, duration	Pharmacist-driven ASP significantly improved antibiotic appropriateness; it also reduced mortality risk
Sirijatuphat et al. [47]	Feasibility, Challenges, and Benefits of Global Antimicrobial Resistance Surveillance System Implementation: Results from a Multicenter Quasi-Experimental Study	Surveillance	GLASS in provincial hospitals	AMR patterns, CAI vs HAI classification	Feasible but challenging; higher resistance in HAI; useful for local guidelines
Wangchinda et al. [48]	Impact of Antibiotic Authorisation at Three Provincial Hospitals in Thailand: Results from a Quasi-Experimental Study	Antimicrobial stewardship	Antibiotic authorization in provincial hospitals	Antibiotic DOT, clinical response, LOS	Targeted antibiotic decreases DOT and LOS; increases favorable response; feasible in resource-limited settings
Thomnoi et al. [49]	Impact of Pharmacist-Led Implementation of a Community Hospital-Based Outpatient Parenteral Antimicrobial Therapy on Clinical Outcomes in Thailand	Antimicrobial stewardship	Pharmacist-led OPAT monitoring with CPG	Appropriate dosing, lab monitoring, outcomes	Dosing appropriateness increased to 100%; unfavorable outcomes reduced (6% vs 26%); demonstrated feasibility and benefit of pharmacist-led CohPAT in a community hospital setting
Loo et al. [50]	Antimicrobial Stewardship in Cardiac Device Surgery: Impact of Behavioural Change Interventions on Extended Prophylaxis Practices	Antimicrobial stewardship	Behavioral ASP bundle for cardiac device surgery	Extended prophylaxis use, SSI rates	Significantly reduced unnecessary extended antibiotic prophylaxis from 43.8% to 24.0%; no SSI increase
Aoybamroong et al. [51]	Impact of an Antibiotic Stewardship Program on Antibiotic Prescription for Acute Respiratory Tract Infections in Children: A Prospective Before-After Study	Antimicrobial stewardship	Guideline dissemination & feedback for pediatric ARTIs	Appropriate antibiotic prescribing	Appropriateness increased from 77.5% to 83.4%; overprescribing reduced; Greatest improvement among faculty staff
Chautrakarn et al. [52]	Impact of a Prospective Audit and Feedback Antimicrobial Stewardship Program in Pediatric Units in Tertiary Care Teaching Hospital in Thailand	Antimicrobial stewardship	PAF for vancomycin, colistin, meropenem in pediatrics	Antibiotic DOT, LOS, mortality	Vancomycin & colistin use reduced; no LOS/mortality change; safe in pediatrics
Huong et al. [53]	Assessing feasibility of establishing antimicrobial stewardship programmes in two provincial- level hospitals in Vietnam: an implementation research study	Antimicrobial stewardship	Participatory AMS establishment in provincial hospitals	Feasibility, antibiotic use, process indicators	AMS was feasible; PAF implemented; variability in antibiotic use observed
Waleekhachonloet et al. [54]	Effects of a national policy advocating rational drug use on decreases in outpatient antibiotic prescribing rates in Thailand	Policy—regulations /Antimicrobial stewardship	National RDU policy with targets & feedback	Antibiotic prescribing rates (%) for respiratory infections, acute diarrhea, fresh wounds	Reduced prescribing rates for respiratory infections, diarrhea, and wounds; targets were nearly met
Arulappen et al. [55]	The Impact of Antimicrobial Stewardship Program on Injudicious Use of Cefuroxime	Antimicrobial stewardship	AMS for surgical prophylaxis (guideline + restriction)	Cefuroxime use, guideline compliance, SSI	Cefuroxime use reduced; compliance increased to 94.3%; SSI reduced from 17 to 9%
Wai et al. [56]	Community-based MDR-TB care project improves treatment initiation in patients diagnosed with MDR-TB in Myanmar	Capacity Building /Antimicrobial Stewardship	Community-based MDR-TB support package	Treatment initiation, time to initiation	Increased treatment initiation and reduced time to treatment
Althaus et al. [57]	Effect of point-of-care C-reactive protein testing on antibiotic prescription in febrile patients attending primary care in Thailand and Myanmar: an open-label, randomised, controlled trial	Capacity Building /Antimicrobial Stewardship	CRP POCT with thresholds in primary care	Antibiotic prescribing rates	A small but statistically significant reduction in antibiotic use
Thamlikitkul et al. [58]	Integrated one-day surveillance of antimicrobial use, antimicrobial consumption, antimicrobial resistance, healthcare-associated infection, and antimicrobial resistance burden among hospitalized patients in Thailand	Surveillance	Integrated one-day surveillance (AMU, AMR, HAI)	AMU, AMR, HAI prevalence, burden	Feasible; AMU prevalence was 51.5%; HAI prevalence was 14.0%; AMR associated with longer stays and higher costs
Karyanti et al. [59]	Evaluation of educational intervention program on appropriate antimicrobial usage in department of child health, Indonesia	Antimicrobial stewardship /Awareness/Capacity Building	Educational intervention for pediatric prescribing	Appropriate antimicrobial use (Gyssens)	Appropriate use increased from 58 to 67%; educational intervention had a positive but modest effect
Nguyen-Thi et al. [60]	Impact of Antimicrobial Stewardship Program on Vancomycin Usage: Costs and Outcomes at Hospital for Tropical Diseases in Ho Chi Minh City, Vietnam	Antimicrobial Stewardship	Enhanced ASP with stricter policies for vancomycin	Clinical outcomes, treatment cost	Enhanced ASP reduced mortality, infection recurrence, and treatment costs in vancomycin use
Thong et al. [61]	Impact of targeted educational intervention towards public knowledge and perception of antibiotic use and resistance in the state of Perak, Malaysia	Awareness	Pharmacist-delivered educational leaflet on AMR	Knowledge & perception scores	Significantly improved knowledge and perception on antibiotic use, with sustained effects over two weeks
Phan et al. [62]	Impact of antimicrobial stewardship intervention in clean and clean- contaminated surgical procedures at a Vietnamese national hospital	Antimicrobial stewardship	Multifaceted ASP for surgical prophylaxis	Appropriateness, duration, cost	Appropriateness increased to 39%; postoperative prophylaxis duration and cost reduced
Yong et al. [63]	Utilising a COM-B framework to modify antibiotic prescription behaviours following third molar surgeries	Antimicrobial stewardship	COM-B-based intervention for dental antibiotic use	Prescription rates post third molar surgery	Prescription reduced from 84.45% to 20.89%; without increasing complications
Woo et al. [64]	An evaluation of the intravenous to oral antimicrobial conversion program in the inpatient setting	Antimicrobial stewardship	IV-to-PO conversion program with pharmacist training	Conversion rate, acceptance	Training alone did not improve IV-to-PO conversion or pharmacist intervention rates, indicating need for stronger AMS strategies
Liew et al. [65]	Antimicrobial stewardship programme: a vital resource for hospitals during the global outbreak of coronavirus disease 2019 (COVID-19)	Antimicrobial stewardship /Program adaptation & maintenance	ASP adaptation during COVID-19 (tele-auditing)	Broad-spectrum antibiotic use, ASP workload	Broad-spectrum use increased by 25.5% during the early pandemic; while ASP reduced treatment duration and hospital stay and remain essential to guide prescribing
Bâtie et al. [66]	Understanding the implementation of antimicrobial resistance policies in Vietnam: a multilayer analysis of the veterinary drug value chain	Policy—regulations /Awareness/Capacity Building	Analysis of veterinary antibiotic policy implementation	Stakeholder understanding, barriers	Barriers: lack of local involvement and informal distribution
Lota et al. [67]	A Qualitative Study on the Design and Implementation of the National Action Plan on Antimicrobial Resistance in the Philippines	Policy – Regulation /Intervention	Qualitative study on Philippine NAP implementation	Enabling factors, challenges, One Health integration	Progress in human health; environment sector lagging; need resources and coordination
Sumpradit et al. [68]	Thailand's national strategic plan on antimicrobial resistance: progress and challenges	Policy – Regulation/Surveillance/Infection Prevention and Control/Awareness/Antimicrobial Stewardship	Thailand's National Strategic Plan on AMR	Progress indicators (consumption, AMR)	Animal antibiotic use reduced 49%; human use increased; surveillance established; funding variable
Bordier et al. [69]	Antibiotic resistance in Vietnam: moving towards a One Health surveillance system	Policy—Regulation/Surveillance	One Health surveillance system mapping in Vietnam	Stakeholder mapping, implementation factors	System silos hinder One Health collaboration; seven key factors impede it
Sirijatuphat et al. [70]	Implementation of global antimicrobial resistance surveillance system (GLASS) in patients with bacteremia	Surveillance	GLASS for bacteremia	True infection rate, AMR, mortality, cost	Only 15.2% were true infections; resistant bacteremia was associated with higher mortality and costs
Drabarek et al. [71]	Examining the challenges in sustaining user engagement with a mobile app to enhance multidrug-resistant tuberculosis (MDR-TB) care in Vietnam and its implications for implementing person-centred mHealth interventions	Capacity Building	mHealth app for MDR-TB care (Bac Sy Minh)	App engagement, user patterns, HCW workload	Engagement dropped sharply; the app increased HCW workload, highlighting challenges in sustaining engagement
Swe et al. [72]	Evaluation of the forum theatre approach for public engagement around antibiotic use in Myanmar	Awareness	Forum theatre for AMR public engagement in Myanmar	Knowledge change, audience engagement	Improved knowledge; high enjoyment; preferred over traditional health talks
Phan et al. [73]	Sustained effects of a multimodal campaign aiming at hand hygiene improvement on compliance and healthcare-associated infections in a large gynaecology/obstetrics tertiary-care centre in Vietnam	Surveillance/Awareness/Capacity Building/Infection Prevention and Control	Multimodal hand hygiene campaign (9 years)	Hand hygiene compliance, HAI rates	Compliance increased from 21.5% to 75.1%; HAI reduced; SSI trends varied
Argimón et al. [74]	Integrating whole-genome sequencing within the National Antimicrobial Resistance Surveillance Program in the Philippines	Surveillance	Integrated WGS into Philippine AMR surveillance; sequenced 805 carbapenem-resistant isolates from 2013–2014	Genetic lineages, resistance mechanisms, plasmid distribution, transmission patterns	WGS revealed horizontal resistance spread, plasmid-driven NICU outbreak, and international high-risk clones, prompting infection control interventions
Anugulruengkitt et al. [75]	Barriers in implementing antibiotic stewardship programmes at paediatric units in academic hospitals in Thailand: a qualitative study	Antimicrobial Stewardship	Qualitative study exploring barriers to AMS implementation in Thai pediatric units using the CFIR framework	Themes related to AMS implementation barriers and facilitators; ranked preferences for AMS interventions	Barriers: hierarchy, fear, resources, lack of authority. Facilitators: motivation, collaboration, education. Top interventions: de-escalation, guidelines, antibiograms, audit
Cheng et al. [76]	Antimicrobial surface coating in the emergency department as protective technology for infection control (ASEPTIC): a pilot randomized controlled trial	Infection prevention and control	Double-blind, placebo-controlled RCT of NOMOBAC antimicrobial surface coating vs. placebo saline applied to 96 stretcher rails in Singapore emergency department; bacterial sampling at 24 h, 7 days, and 180 days post-application	Total aerobic bacterial contamination (CFU/cm^2^)	Significant 24 h bacterial reduction (0.61 vs. 1.01 CFU/cm^2^); effect lost by 7 days due to coating wear; 28.1% of rails exceeded baseline threshold
Lim et al. [77]	Automating the Generation of Antimicrobial Resistance Surveillance Reports: Proof-of-Concept Study Involving Seven Hospitals in Seven Countries	Surveillance	Developed and tested AMASS software to automate AMR surveillance reporting; implemented in 7 hospitals across 7 countries; ASEAN sites included Cambodia, Laos, Myanmar, Thailand, Vietnam	Feasibility, time to generate reports, compatibility with different data systems, detection of resistance variation	AMASS successfully generated standardized WHO GLASS-compliant reports in 1–3 min; detected local variation in AMR prevalence across ASEAN sites; resistance rates for 3GC-resistant E. coli bacteremia ranged from 19% (Vietnam) to 85% (Cambodia); tool was compatible with WHONET and various laboratory information systems

Acute respiratory tract infection (ARTI); Antimicrobial resistance Surveillance System (AMASS); antimicrobial stewardship programme (ASP); antimicrobial use (AMU); Capability, Opportunity, Motivation-Behaviour model (COM-B); C-reactive-protein point-of-care testing (CRP POCT); Clostridium difficile infection (CDI); community-acquired infection (CAI); Community Hospital-based Parenteral Anti-infective Therapy (CohPAT); computerised decision support system (CDSS); Days of Therapy (DOT); Defined Daily Dose (DDD); extended-spectrum beta-lactamase (ESBL); focus group discussions (FGDs); Clinical Practice Guidelines (CPG); Global Antimicrobial Resistance Surveillance System (GLASS); hospital-associated infection (HAI); healthcare worker (HCW); information technology (IT); intravenous to oral (IV-to-PO); Knowledge, attitude, perception (KAP); length of hospital stay (LOS); Length of Therapy (LOT); mobile health; lot quality assurance sampling (LQAS); (mHealth); Multidrug-resistant tuberculosis (MDR-TB) Outpatient Parenteral Antimicrobial Therapy (OPAT); Philippine National Action Plan (PNAP) national action plan (NAP); prospective audit with feedback (PAF); randomized controlled trial (RCT); rational drug use (RDU); Surgical Site Infection (SSI).

### Synthesis of overall findings

Taken together, the 57 included studies reveal several clear patterns in the ASEAN AMR intervention landscape. First, antimicrobial stewardship programs in hospital settings dominated the evidence base, with 36 studies (63.2%) incorporating stewardship components. These interventions consistently reported improvements in antibiotic consumption, prescribing appropriateness, duration of therapy, or cost savings. Second, evidence was heavily concentrated in three countries, Thailand, Singapore, and Vietnam, which together accounted for 70.2% of all studies. In contrast, no eligible peer-reviewed studies were identified from Brunei Darussalam, and Laos and Cambodia were represented only through disaggregated data from one multi-country surveillance initiative [77]. Third, the human health sector accounted for 86.0% of studies, with only five studies (8.8%) adopting a multisectoral One Health approach and no studies addressing the environmental sector in isolation. Among non-hospital settings, interventions included multi-setting studies (n = 3), community-based programs (n = 2), one mixed healthcare facility/community study (n = 1), farm-level interventions (n = 1), veterinary clinic initiatives (n = 1), one national-level study (n = 1), and one study without explicit setting specification (n = 1). Fourth, observational designs predominated (50.9%), with quasi-experimental designs in 22.8% of studies and experimental designs in 10.5%. Fifth, outcomes measured were predominantly short-term process indicators, including defined daily doses, duration of therapy, prescribing rates, and costs. Finally, no study referenced an established implementation science framework, and only four studies (7.0%) employed mixed-methods approaches.

## Discussion

This scoping review revealed that antimicrobial resistance interventions in ASEAN have achieved meaningful improvements in antibiotic use and prescription quality, particularly within hospital-based antimicrobial stewardship programs. The included studies consistently demonstrated reductions in antibiotic consumption, with documented decreases in defined daily doses, shorter durations of therapy, and substantial cost savings (Table 3). Improvements in prescribing appropriateness were also evident, with several studies reporting significant increases in guideline adherence and reductions in inappropriate antibiotic use. However, these gains remain largely localized and have not consistently translated into sustained reductions in antimicrobial resistance. This disconnect reflects deeper structural challenges, including sectoral fragmentation, limited theoretical grounding, and weak integration across the One Health domains. Collectively, these findings highlight a regionally active but methodologically fragmented AMR response, underscoring the need to move beyond isolated, facility-based successes toward more integrated, theory-informed, and system-level strategies to combat AMR.

The first finding is the absolute inequity in research and intervention focus across the ASEAN region. Over 70% of the identified studies originated from three countries: Thailand (26.3%), Singapore (22.8%), and Vietnam (21.1%). In contrast, Myanmar and Malaysia were significantly underrepresented. Moreover, no eligible peer-reviewed studies were identified from Brunei Darussalam in this review. Laos and Cambodia were represented only through disaggregated surveillance data from a single multi-country initiative [77]. This difference likely reflects a complex interaction of factors, including variations in research capacity, healthcare infrastructure, and funding availability [11]. The underrepresentation of certain countries creates a critical evidence gap, as AMR intervention research in low-resource and fragile health systems is often constrained by limited surveillance capacity and evaluative infrastructure, thereby obscuring context-specific challenges and hindering the development of region-wide strategies[78].

Similarly, the overwhelming focus on the human health sector (86.0% of studies) and hospital settings (82.5%) underlines a persistent blind spot toward the animal and environmental pillars of One Health. Only five studies (8.8%) adopted an explicit multisectoral approach. Among the studies conducted outside hospital settings, interventions included multi-setting interventions (n = 3), community-based programs (n = 2), one mixed healthcare facility/community study (n = 1), farm-level interventions (n = 1), veterinary clinic initiatives (n = 1), one national-level study (n = 1), and one study without explicit setting specification (n = 1). The sole national-level study, Vietnam's One Health surveillance system mapping [69], represented an important example of government-led policy engagement. This sectoral siloing is a major impediment to tackling AMR at its source, as evidenced by studies highlighting the lack of formal environmental sector engagement and weak local implementation of national policies [39, 67]. Antimicrobial use in livestock and aquaculture is a major driver of resistance in Southeast Asia [79, 80]; however, only three studies addressed this sector. The complete lack of environmental studies, despite recognized risks from pharmaceutical waste, agricultural runoff, and wastewater contamination [81], represents a fundamental gap in operationalizing One Health.

Our synthesis indicates that well-structured antimicrobial stewardship (AMS) programs in hospital settings are demonstrably effective in improving processes and economic outcomes. Interventions employing prospective audit and feedback (PAF), formulary restriction (pre-authorization), and guideline implementation consistently led to significant reductions in antibiotic consumption [45], shorter durations of therapy, lower costs, and improved prescribing appropriateness without compromising clinical safety [22, 41, 55]. The largest evaluation in our review, a nationwide mixed-methods study of 575 Indonesian hospitals, found that successful implementation depended on strong leadership support, dedicated multidisciplinary teams, and integration into routine workflows [40]. Hospitals with higher leadership support scores achieved significantly better ASP implementation, underscoring the importance of organizational capacity [33]. These effects appear to be mediated by the organizational conditions under which they are implemented, including strong institutional leadership, dedicated multidisciplinary teams, and the routine use of local prescribing data embedded within clinical workflows. In the ASEAN context, this pattern suggests that stewardship effectiveness is less a function of intervention type alone and more contingent on underlying organizational capacity, governance structures, and accountability mechanisms that enable sustained integration into routine care [82].

Conversely, interventions that failed to achieve their primary aims consistently reflected structural and contextual misalignment. Programs with limited intensity, weak integration into routine practice, or insufficient adaptation to local prescribing realities showed attenuated effects on the primary outcome. For instance, an enhanced pediatric ASP in Vietnam improved antibiotic selection (increased access to antibiotics) but did not reduce overall consumption, highlighting the challenge of changing entrenched prescribing habits for severe infections [27]. A cluster-RCT in Vietnamese district hospitals achieved only a modest 6.3% reduction in inappropriate prescribing, underscoring that foundational AMS activities (committees, guidelines) are necessary but insufficient against a backdrop of extremely high baseline misuse [37]. In the animal sector, a farm-level intervention saw a dramatic drop in colistin use due to a concurrent national ban, but no reduction in AMR prevalence, illustrating the complex ecology of resistance and the limits of standalone technical training without broader system changes [28]. Collectively, these findings imply that AMS interventions require sufficient intensity, contextual responsiveness, and alignment with broader regulatory and health system reforms to generate meaningful and sustained impact [83].

Surveillance interventions effectively detect resistance patterns and identify outbreaks; however, they rarely achieve a direct clinical impact unless linked to actionable feedback. Ginting et al. (2019) demonstrated that LQAS could identify high-resistance populations cost-effectively, with > 98% sensitivity at US$466 per site, offering a feasible approach for resource-limited settings [26]. Another study showed that integrating whole-genome sequencing into the Philippine national surveillance program could trigger infection control responses, identifying a plasmid-driven NICU outbreak of *K. pneumoniae* ST340 and international high-risk clones of *E. coli* ST410 carrying both *bla*NDM-1 and *bla*OXA-181 [74]. These examples demonstrate that surveillance can be an active intervention catalyst when linked to action, and not merely a passive monitoring tool.

Interventions incorporating behavioral insights have shown significant potential. A COM-B model-based intervention in dentistry reduced unnecessary antibiotic prescriptions by over 60% [63], and a "nudge" intervention leveraging peer influence from an ASP champion successfully reduced extended surgical prophylaxis [50]. In the veterinary sector, prescribing decisions are reported to be heavily influenced by business considerations and client relationships, with veterinarians expressing concern about losing clients if they refuse to prescribe antibiotics, representing a behavioral barrier that requires targeted intervention [42]. These findings position behaviorally informed strategies as a critical advancement beyond knowledge-based stewardship models [84], as they directly target prescribers’ motivation, capability, and social norms rather than assuming rational guideline adoption [85]. Similarly, innovative public engagement using forum theatre [72] and gamified mobile apps [38] has proven highly effective in improving community knowledge and attitudes, suggesting that participatory, culturally adapted methods outperform traditional didactic approaches.

IPC interventions have demonstrated significant benefits when implemented as multimodal strategies. The first double-blind randomized controlled trial evaluating antimicrobial surface coating in an emergency department found that the NOMOBAC coating reduced bacterial contamination by 40% after 24 h; however, the effect was not sustained owing to physical wear, highlighting the need for improved durability [76]. A nine-year multimodal campaign in Vietnam integrated surveillance of hand hygiene compliance, ongoing staff training and capacity building, and public awareness initiatives alongside core IPC strategies. This comprehensive approach achieved sustained improvement in hand hygiene compliance from 21.5% to 75.1%, demonstrating that long-term commitment to integrated IPC strategies can yield lasting results [73].

Notably, 15 studies (26.8%) evaluated multi-component interventions that combined two or more strategies. Common combinations included antimicrobial stewardship combined with awareness, policy, or surveillance. For example, Thailand’s National Strategic Plan combined policy, surveillance, and antimicrobial stewardship components and achieved a 49% reduction in antimicrobial use in animals. These multi-component approaches showed particular promise, suggesting that coordinated strategies addressing multiple drivers simultaneously may be more effective than single-component interventions. However, their heterogeneity limits generalizable conclusions about the optimal combination [68].

Persistent barriers to implementation emerged across multiple studies. Resource limitations, including inadequate staffing, lack of dedicated antimicrobial stewardship personnel, and insufficient microbiology capacity, were repeatedly cited [40, 53]. In Thai pediatric units, junior staff described reluctance to question prescribing decisions made by senior attending physicians, citing respect for clinical hierarchy and perceived experience differentials as barriers to antimicrobial stewardship implementation [75]. This cultural barrier, deeply rooted in Asian medical hierarchies, requires targeted interventions that address authority dynamics rather than simply providing education [86, 87].

A critical observation is that while many interventions have successfully improved antibiotic use metrics and even clinical outcomes, direct evidence of their impact on reducing AMR incidence or prevalence remains sparse and equivocal. Longitudinal data from Singapore associated a decade of national ASP efforts with decreasing trends in resistant organisms[23], and mathematical modeling projected that Thailand's National Strategic Plan could reduce human colonization with resistant bacteria by 6.0–18.8% over 20 years [34]. However, most studies were either not designed or lacked the duration to measure such downstream effects. This pattern reflects a fundamental challenge in AMR control: resistance is a slowly evolving, system-level outcome shaped by cumulative and interacting selective pressures that extend beyond hospital antibiotic use alone [88]. In this context, the limited incorporation of integrated One Health strategies in the reviewed interventions suggests that current efforts address only a partial component of the ecological drivers of resistance, thereby constraining their capacity to generate measurable reductions in AMR at the population level [33].

The methodological landscape of the included studies reveals limitations that constrain the strength of the evidence. The predominance of observational designs (50.0%) and the lack of control groups in many evaluations introduce significant risks of bias, making causal attribution difficult to determine. Among the evaluation studies, the outcomes were heterogeneous, often focusing on short-term process measures rather than long-term clinical or resistance outcomes.

Crucially, our review identified a near-total absence of explicit theoretical frameworks to guide intervention design or evaluation. No study referenced established implementation science theories, such as the CFIR [89], TDF, or complex intervention frameworks, such as the MRC guidelines. This theoretical paucity constrains explanatory depth, as interventions are evaluated primarily on whether they work, rather than why they succeed or fail within specific organizational and sociocultural contexts. Consequently, the current evidence base remains fragmented and difficult to generalize, limiting its utility for informing scalable and context-sensitive AMR interventions across diverse settings.

Building on the identified fragmentation, sectoral imbalance, and methodological limitations of AMR interventions in ASEAN, future efforts must transcend these limitations with region-specific strategies. First, given that over 70% of evidence originated from just three countries (Thailand, Singapore, Vietnam) while Brunei, Laos, and Cambodia had minimal or no country-specific studies, the ASEAN Secretariat should establish a dedicated AMR research fund targeting evidence-gap countries. This fund could operate through twinning arrangements pairing established AMR research centers in Thailand, Singapore, or Vietnam with institutions in underrepresented member states, explicitly prioritizing capacity-building deliverables alongside research outputs. Second, given that 86.0% of identified studies focused on human health and no studies addressed the environmental sector in isolation, ASEAN member states should mandate environmental sector representation in their national AMR governance committees and require environmental monitoring components in all nationally funded AMR projects. Third, the region would benefit from an ASEAN-wide implementation science training initiative to build capacity in theory-informed intervention design and evaluation. This initiative, potentially hosted by the ASEAN Centre for Public Health Emergencies and Emerging Diseases (ACPHEED), would directly address our finding that no study referenced established implementation science frameworks. Fourth, existing GLASS implementation sites in Thailand could serve as regional training hubs for surveillance capacity building in Cambodia, Laos, and Myanmar, with structured exchange programs for laboratory personnel and epidemiologists. Without such targeted, ASEAN-specific advances, AMR initiatives are likely to continue producing short-term improvements in antimicrobial use without achieving sustained population-level resistance reduction.

This scoping review provides a comprehensive and regionally focused synthesis of antimicrobial resistance (AMR) interventions implemented across the ASEAN region, spanning stewardship, surveillance, policy, and educational approaches across diverse sectors and settings. A key strength is its explicit attention to geographical and sectoral distribution, enabling the identification of structural inequities in the evidence base, particularly the concentration of evaluative studies in higher-capacity countries and hospital-based human health settings, alongside limited representation of animal and environmental sectors. The inclusion of diverse study designs, ranging from randomized controlled trials to qualitative implementation research, enabled a comprehensive understanding of both intervention effectiveness and the contextual factors influencing implementation success. The inclusion of recently published studies up to 2025 ensures that the findings reflect the most current evidence. By mapping intervention outcomes and organizational, behavioral, and theoretical characteristics, this review moves beyond effectiveness alone to offer explanatory insights into how and under what conditions AMR interventions are implemented in the region.

However, this study has several limitations that should be acknowledged. As a scoping review, no formal risk-of-bias assessment was conducted, limiting causal inferences regarding intervention effectiveness. The predominance of observational designs, short follow-up periods, and heterogeneous outcome measures constrained comparability and precluded quantitative syntheses. In addition, reliance on the published literature may have led to the underrepresentation of ongoing but unevaluated interventions, particularly in lower-resource or fragile settings. Furthermore, this review was limited to peer-reviewed literature. Many AMR interventions in ASEAN countries are implemented and evaluated by national governments, non-governmental organizations, and multilateral agencies (e.g., WHO, FAO, WOAH), with results disseminated through unpublished reports, policy briefs, or organizational websites. For instance, the FAO has supported AMR surveillance initiatives in Cambodia's livestock sector under the ACT project [90], and the WHO Regional Office for South-East Asia regularly publishes AMR progress reports and roadmaps that fall outside peer-reviewed channels [91]. Similarly, the Vietnamese Department of Animal Health and Production, in collaboration with FAO Vietnam, implemented the Farmer School Community initiative, which demonstrated reduced antibiotic use and cost in poultry farming through targeted farmer training and improved husbandry and biosecurity practices [92]. This introduces potential publication bias and may underestimate intervention activity, particularly in countries with less developed academic publishing infrastructure such as Laos, Cambodia, and Myanmar. Future mapping efforts should systematically incorporate grey literature sources and government repositories to provide a more complete picture of the ASEAN AMR intervention landscape. Additionally, 25 records (22.0% of those sought for full-text review) were excluded due to full-text unavailability after database and institutional access screening. Moreover, formal inter-rater reliability statistics (e.g., Cohen's kappa) were not prospectively calculated during the screening process. However, all disagreements were resolved through consensus discussion or third-reviewer adjudication, and a pre-screening calibration exercise was conducted to ensure consistent application of eligibility criteria. Moreover, our data extraction did not systematically capture certain intervention characteristics that would enhance mapping exercises, including exact implementation periods, scale (local, regional, national), and institutional type (e.g., government-led, hospital-led, NGO-led). Future reviews should incorporate these dimensions to provide a more granular understanding of the AMR intervention landscape in ASEAN. Finally, the apparent absence of explicit theoretical frameworks may partly reflect underreporting rather than a complete lack of theory use, although this nonetheless limits the interpretability and transferability of the findings.

## Conclusion

This scoping review demonstrates that ASEAN countries have implemented a broad range of antimicrobial stewardship, surveillance, policy, and educational interventions that can improve antibiotic use and selected health system outcomes. However, the current evidence base remains fragmented, geographically uneven, and heavily concentrated within hospital-based health settings, with limited incorporation of integrated One Health approaches in the field. The findings indicate that the effectiveness and scalability of AMR interventions in ASEAN are shaped not only by intervention type but also by organizational capacity, governance arrangements, behavioral mechanisms, and cross-sectoral coordination. Moving forward, AMR control in the region requires a strategic shift toward theory-informed implementation research, strengthened evaluative capacity in underrepresented settings, and the use of surveillance systems as active instruments for action rather than passive monitoring tools. Without such advances, AMR initiatives are likely to continue producing short-term improvements in antimicrobial use without achieving sustained population-level resistance reduction.

## Supplementary Information

Supplementary Material 1.

## Data Availability

No datasets were generated or analysed during the current study.
