# Supplementary material for: Mapping antimicrobial resistance interventions in ASEAN countries: a scoping review of implementation patterns, One Health integration, and evidence gaps
Source: Trop Med Health. 2026 May 30;54:104. doi: 10.1186/s41182-026-00992-w (PMC13227827; doi:10.1186/s41182-026-00992-w)
Supplement: Supplementary file 1 — Supplementary Material 1. [file 41182_2026_992_MOESM1_ESM.pdf]

Part A: Preferred Reporting Items for Systematic reviews and Meta-Analyses extension for Scoping Reviews (PRISMA-ScR) Checklist

SECTION	ITEM	PRISMA-ScR CHECKLIST ITEM	REPORTED ON PAGE #
**TITLE**			
Title	1	Identify the report as a scoping review.	1
**ABSTRACT**			
Structured summary	2	Provide a structured summary that includes (as applicable): background, objectives, eligibility criteria, sources of evidence, charting methods, results, and conclusions that relate to the review questions and objectives.	2
**INTRODUCTION**			
Rationale	3	Describe the rationale for the review in the context of what is already known. Explain why the review questions/objectives lend themselves to a scoping review approach.	4
Objectives	4	Provide an explicit statement of the questions and objectives being addressed with reference to their key elements (e.g., population or participants, concepts, and context) or other relevant key elements used to conceptualize the review questions and/or objectives.	4
**METHODS**			
Protocol and registration	5	Indicate whether a review protocol exists; state if and where it can be accessed (e.g., a Web address); and if available, provide registration information, including the registration number.	5
Eligibility criteria	6	Specify characteristics of the sources of evidence used as eligibility criteria (e.g., years considered, language, and publication status), and provide a rationale.	6
Information sources*	7	Describe all information sources in the search (e.g., databases with dates of coverage and contact with authors to identify additional sources), as well as the date the most recent search was executed.	5
Search	8	Present the full electronic search strategy for at least 1 database, including any limits used, such that it could be repeated.	5
Selection of sources of evidence†	9	State the process for selecting sources of evidence (i.e., screening and eligibility) included in the scoping review.	6-7
Data charting process‡	10	Describe the methods of charting data from the included sources of evidence (e.g., calibrated forms or forms that have been tested by the team before their use, and whether data charting was done independently or in duplicate) and any processes for obtaining and confirming data from investigators.	7
Data items	11	List and define all variables for which data were sought and any assumptions and simplifications made.	7

SECTION	ITEM	PRISMA-ScR CHECKLIST ITEM	REPORTED ON PAGE #
Critical appraisal of individual sources of evidence§	12	If done, provide a rationale for conducting a critical appraisal of included sources of evidence; describe the methods used and how this information was used in any data synthesis (if appropriate).	7
Synthesis of results	13	Describe the methods of handling and summarizing the data that were charted.	7
**RESULTS**			
Selection of sources of evidence	14	Give numbers of sources of evidence screened, assessed for eligibility, and included in the review, with reasons for exclusions at each stage, ideally using a flow diagram.	7-9
Characteristics of sources of evidence	15	For each source of evidence, present characteristics for which data were charted and provide the citations.	15-32
Critical appraisal within sources of evidence	16	If done, present data on critical appraisal of included sources of evidence (see item 12).	7
Results of individual sources of evidence	17	For each included source of evidence, present the relevant data that were charted that relate to the review questions and objectives.	15-32
Synthesis of results	18	Summarize and/or present the charting results as they relate to the review questions and objectives.	33
**DISCUSSION**			
Summary of evidence	19	Summarize the main results (including an overview of concepts, themes, and types of evidence available), link to the review questions and objectives, and consider the relevance to key groups.	33-38
Limitations	20	Discuss the limitations of the scoping review process.	38
Conclusions	21	Provide a general interpretation of the results with respect to the review questions and objectives, as well as potential implications and/or next steps.	38-39
**FUNDING**			
Funding	22	Describe sources of funding for the included sources of evidence, as well as sources of funding for the scoping review. Describe the role of the funders of the scoping review.	39

Part B: Search Strategy

Step	Tools	Output
Generating synonym	MesH	(("antimicrobial resistance" OR "antibiotic resistance" OR "drug resistance, microbial"<br>OR "antimicrobial stewardship" OR "antibiotic stewardship" OR AMR OR MDR<br>OR "antibiotic misuse" OR "antimicrobial misuse" OR "antimicrobial consumption")<br>AND<br>(intervention* OR program* OR policy OR policies OR strategy OR strategies<br>OR initiative* OR campaign* OR "education program*" OR "stewardship program*"<br>OR "infection prevention and control" OR IPC OR "surveillance system*"<br>OR "behavioral intervention*" OR "behaviour change" OR "public health intervention*")<br>AND<br>("Southeast Asia" OR ASEAN OR "Association of Southeast Asian Nations"<br>OR Indonesia OR Malaysia OR Singapore OR Thailand OR Philippines OR "the Philippines"<br>OR Vietnam OR "Viet Nam" OR Laos OR "Lao PDR" OR Cambodia OR Kampuchea<br>OR Myanmar OR Burma OR Brunei OR "Brunei Darussalam"))
Searching related articles	Pubmed (26 Nov)<br>Filters applied: From 2018 to 2025, Classical Article, Clinical Trial, Randomized Controlled Trial, Randomized Controlled Trial, Veterinary.<br><br>Filters applied: Clinical Trial, Comparative Study,	1. ("Antimicrobial Resistance"[MeSH Terms] OR "antimicrobial resistance"[tiab] OR "antibiotic resistance"[tiab] OR "drug resistant"[tiab]) AND (intervention*[tiab] OR program*[tiab] OR polic*[tiab] OR "behavior change"[tiab] OR "public health intervention"[tiab] OR "health system"[tiab]) AND (ASEAN[tiab] OR "Southeast Asia"[tiab] OR "South-East Asia"[tiab] OR Indonesia[tiab] OR Malaysia[tiab] OR Thailand[tiab] OR Vietnam[tiab] OR Cambodia[tiab] OR Laos[tiab] OR Myanmar[tiab] OR Singapore[tiab] OR Brunei[tiab]) → 11<br>2. ("antimicrobial resistance" OR "antibiotic resistance" OR "drug resistance") AND (intervention* OR program* OR policy OR policies OR "public health" OR "health system") AND ("Southeast Asia" OR ASEAN OR Indonesia OR Malaysia OR Thailand OR Vietnam OR Cambodia OR Laos OR Myanmar OR Singapore OR Brunei) → 58<br>3. ("infection control" OR "infection prevention") AND ("antimicrobial resistance" OR "antibiotic resistance") AND (Indonesia OR Malaysia OR Thailand OR Vietnam OR Cambodia OR Laos OR Myanmar OR Singapore OR Brunei) → 5<br>4. ("Antimicrobial Stewardship"[MeSH Terms] OR "antibiotic stewardship"[tiab] OR ASP[tiab] OR "antibiotic use"[tiab] OR "antibiotic prescribing"[tiab]) AND (intervention*[tiab] OR program*[tiab] OR implementation[tiab] OR effectiveness[tiab] OR evaluation[tiab]) AND ("Southeast Asia"[tiab] OR Indonesia[tiab] OR Malaysia[tiab] OR Thailand[tiab] OR Vietnam[tiab] OR Cambodia[tiab] OR Laos[tiab] OR Myanmar[tiab] OR Singapore[tiab] OR Brunei[tiab]) → 6<br>5. ("Antimicrobial Resistance"[MeSH Terms] OR "antimicrobial resistance"[tiab]) AND ("National Action Plan"[tiab] OR policy[tiab] OR regulation[tiab] OR legislation[tiab] OR governance[tiab]) AND (Indonesia[tiab] OR Malaysia[tiab] OR Thailand[tiab] OR Vietnam[tiab] OR Cambodia[tiab] OR Laos[tiab] OR Myanmar[tiab] OR Singapore[tiab] OR Brunei[tiab]) → 1<br>6. ("antimicrobial resistance"[tiab] OR "antibiotic resistance"[tiab]) AND ("health education"[MeSH Terms] OR "public awareness"[tiab] OR "behavior change"[tiab] OR "community intervention"[tiab]) AND ("Southeast

	Evaluation Study, Observational Study, from 2018 - 2025.	Asia"[tiab] OR Indonesia[tiab] OR Malaysia[tiab] OR Thailand[tiab] OR Vietnam[tiab] OR Cambodia[tiab] OR Laos[tiab] OR Myanmar[tiab] OR Singapore[tiab] OR Brunei[tiab]) → 2<br>7. ("Hospital"[MeSH Terms] OR hospital*[tiab] OR clinical[tiab]) AND ("antimicrobial resistance"[tiab] OR "antibiotic resistance"[tiab]) AND (intervention*[tiab] OR program*[tiab] OR stewardship[tiab] OR "infection control"[tiab]) AND (Indonesia[tiab] OR Malaysia[tiab] OR Thailand[tiab] OR Vietnam[tiab] OR Cambodia[tiab] OR Laos[tiab] OR Myanmar[tiab] OR Singapore[tiab] OR Brunei[tiab]) → 4<br>8. ("antimicrobial resistance"[tiab] OR "antibiotic resistance"[tiab]) AND (effectiveness[tiab] OR impact[tiab] OR evaluation[tiab] OR outcome*[tiab] OR "intervention study"[tiab]) AND ("Southeast Asia"[tiab] OR ASEAN[tiab] OR Indonesia[tiab] OR Malaysia[tiab] OR Thailand[tiab] OR Vietnam[tiab] OR Cambodia[tiab] OR Laos[tiab] OR Myanmar[tiab] OR Singapore[tiab] OR Brunei[tiab]) → 9<br>9. (("antimicrobial resistance" OR "antibiotic resistance" OR AMR OR MDR OR "antimicrobial stewardship") AND (intervention* OR program* OR policy OR strategy OR "infection prevention and control" OR IPC OR "surveillance" OR "education program" OR "behavior change") AND ("Southeast Asia" OR ASEAN OR Indonesia OR Malaysia OR Singapore OR Thailand OR Philippines OR Vietnam OR Laos OR Cambodia OR Myanmar OR Brunei))→ 107<br>10. ("Antimicrobial Resistance"[MeSH Terms] OR "antimicrobial resistance"[tiab] OR "antibiotic resistance"[tiab] OR "drug resistant"[tiab]) AND (intervention*[tiab] OR program*[tiab] OR polic*[tiab] OR "behavior change"[tiab] OR "public health intervention"[tiab] OR "health system"[tiab]) AND (ASEAN[tiab] OR "Southeast Asia"[tiab] OR "South-East Asia"[tiab] OR Indonesia[tiab] OR Malaysia[tiab] OR Thailand[tiab] OR Vietnam[tiab] OR Cambodia[tiab] OR Laos[tiab] OR Myanmar[tiab] OR Singapore[tiab] OR Brunei[tiab])→ 18<br>11. ("infection control" OR "infection prevention") AND ("antimicrobial resistance" OR "antibiotic resistance") AND (Indonesia OR Malaysia OR Thailand OR Vietnam OR Cambodia OR Laos OR Myanmar OR Singapore OR Brunei)→ 12<br>12. ("antimicrobial resistance"[tiab] OR "antibiotic resistance"[tiab]) AND ("health education"[MeSH Terms] OR "public awareness"[tiab] OR "behavior change"[tiab] OR "community intervention"[tiab]) AND ("Southeast Asia"[tiab] OR Indonesia[tiab] OR Malaysia[tiab] OR Thailand[tiab] OR Vietnam[tiab] OR Cambodia[tiab] OR Laos[tiab] OR Myanmar[tiab] OR Singapore[tiab] OR Brunei[tiab])→ 4<br>13. ("Hospital"[MeSH Terms] OR hospital*[tiab] OR clinical[tiab]) AND ("antimicrobial resistance"[tiab] OR "antibiotic resistance"[tiab]) AND (intervention*[tiab] OR program*[tiab] OR stewardship[tiab] OR "infection control"[tiab]) AND (Indonesia[tiab] OR Malaysia[tiab] OR Thailand[tiab] OR Vietnam[tiab] OR Cambodia[tiab] OR Laos[tiab] OR Myanmar[tiab] OR Singapore[tiab] OR Brunei[tiab])→ 8<br>14. "antimicrobial resistance"[tiab] OR "antibiotic resistance"[tiab]) AND (effectiveness[tiab] OR impact[tiab] OR evaluation[tiab] OR outcome*[tiab] OR "intervention study"[tiab]) AND ("Southeast Asia"[tiab] OR ASEAN[tiab] OR Indonesia[tiab] OR Malaysia[tiab] OR Thailand[tiab] OR Vietnam[tiab] OR Cambodia[tiab] OR Laos[tiab] OR Myanmar[tiab] OR Singapore[tiab] OR Brunei[tiab]) → 21<br>15. ("antimicrobial resistance" OR "AMR" OR "drug resistance" OR "antibiotic resistance" OR "antimicrobial stewardship" OR "infection prevention and control" OR "IPC" OR "antibiotic stewardship" OR "stewardship program") AND (intervention* OR program* OR polic* OR strateg* OR campaign* OR "behavior change" OR

		implement* OR evaluation OR effectiveness OR impact) AND (ASEAN OR "Southeast Asia" OR "South-East Asia" OR Indonesia OR Malaysia OR Thailand OR "Viet Nam" OR Vietnam OR Cambodia OR Laos OR "Lao PDR" OR Myanmar OR Singapore OR Brunei) → 326<br>16. ("antibiotic resistance" OR "antimicrobial resistance" OR "multidrug resistant" OR "MDR" OR "ESBL" OR "carbapenem resistant" OR "MRSA" OR "VRE" OR "drug resistant bacteria") AND ("antibiotic stewardship" OR "antimicrobial stewardship" OR "ASP" OR "infection prevention" OR "infection control" OR surveillance OR intervention* OR program* OR polic* OR guidelines) AND (effectiveness OR evaluation OR impact OR outcome*) AND (ASEAN OR "Southeast Asia" OR "South-East Asia" OR Indonesia OR Malaysia OR Thailand OR Vietnam OR "Viet Nam" OR Cambodia OR Laos OR Myanmar OR Singapore OR Brunei) → 144<br>17. ("antimicrobial resistance" OR "antibiotic resistance" OR "drug resistant infection*" OR "AMR") AND ("public health intervention*" OR "health system intervention*" OR "national action plan" OR "AMR surveillance" OR "One Health" OR "behavior change" OR "community intervention" OR "awareness campaign" OR "regulation" OR "policy") AND (effectiveness OR evaluation OR implement* OR impact) AND (ASEAN OR "Southeast Asia" OR "South-East Asia" OR Indonesia OR Malaysia OR Thailand OR Vietnam OR "Viet Nam" OR Cambodia OR Laos OR Myanmar OR Singapore OR Brunei) → 13<br>**TOTAL BEFORE REMOVE DUPLICATE : 749**<br>**TOTAL AFTER REMOVE DUPLICATE : 391**
	Sciencedirect<br>(26 Nov)<br>2018-2025<br>Research article<br>English	1. "antimicrobial resistance" AND "Intervention" AND "Southeast Asia" → 695<br>2. "antimicrobial resistance" AND Program AND "Southeast Asia" → 446<br>3. "antimicrobial resistance" AND Strategy AND "Southeast Asia" NOT "Systematic Review" NOT Review NOT Bibliometric → 83<br>4. "antimicrobial resistance" AND Strategies AND "Southeast Asia" NOT "Systematic Review" NOT Review NOT Bibliometric → 56<br>5. "antimicrobial resistance" AND "intervention" AND "ASEAN" → 14<br>6. "antimicrobial resistance" AND "intervention" AND Indonesia →142<br>7. "antimicrobial resistance" AND "intervention" AND Malaysia → 219<br>8. "antimicrobial resistance" AND "intervention" AND Singapore → 255<br>9. "antimicrobial resistance" AND "intervention" AND Thailand → 412<br>10. "antimicrobial resistance" AND "intervention" AND Philippines → 143<br>11. "antimicrobial resistance" AND "intervention" AND Vietnam → 276<br>12. "antimicrobial resistance" AND "intervention" AND "Viet nam" → 63<br>13. "antimicrobial resistance" AND "intervention" AND (Laos OR "Lao PDR") → 48<br>14. "antimicrobial resistance" AND "intervention" AND (Cambodia OR Kampuchea) → 78<br>15. "antimicrobial resistance" AND "intervention" AND (Myanmar OR Burma) → 73<br>16. "antimicrobial resistance" AND "intervention" AND (Brunei OR "Brunei Darussalam")) → 15<br>17. "antimicrobial resistance" AND Program AND (Indonesia OR Malaysia OR Singapore OR Thailand) NOT Review → 385

		18. "antimicrobial resistance" AND Program AND (Philippines OR "the Philippines" OR Vietnam OR "Viet Nam" OR Laos OR "Lao PDR") NOT Review → 158<br>19. "antimicrobial resistance" AND Program AND (Cambodia OR Kampuchea OR Myanmar OR Burma OR Brunei OR "Brunei Darussalam") NOT Review → 49<br>20. "antimicrobial resistance" AND Strategy AND (Cambodia OR Kampuchea OR Myanmar OR Burma OR Brunei OR "Brunei Darussalam") NOT Review →50<br>21. "antimicrobial resistance" AND Strategy AND (Philippines OR "the Philippines" OR Vietnam OR "Viet Nam" OR Laos OR "Lao PDR") NOT Review → 136<br>22. "antimicrobial resistance" AND Strategy AND (Indonesia OR Malaysia OR Singapore OR Thailand) NOT Review → 379<br>**TOTAL BEFORE DUPLICATE : 5805**<br>**TOTAL AFTER DUPLICATE : 2219**
	Scopus<br>2018-2025<br>Research article<br>English	1. (("antimicrobial resistance" OR "antibiotic resistance" OR AMR OR MDR OR "antimicrobial stewardship") AND (intervention* OR program* OR policy OR strategy OR "infection prevention and control" OR IPC OR "surveillance" OR "education program" OR "behavior change") AND ("Southeast Asia" OR ASEAN OR Indonesia OR Malaysia OR Singapore OR Thailand OR Philippines OR Vietnam OR Laos OR Cambodia OR Myanmar OR Brunei)) → 1176<br>2. ("antimicrobial resistance" OR "AMR" OR "drug resistance" OR "antibiotic resistance" OR "antimicrobial stewardship" OR "infection prevention and control" OR "IPC" OR "antibiotic stewardship" OR "stewardship program") AND (intervention* OR program* OR polic* OR strateg* OR campaign* OR "behavior change" OR implement* OR evaluation OR effectiveness OR impact) AND (ASEAN OR "Southeast Asia" OR "South-East Asia" OR Indonesia OR Malaysia OR Thailand OR "Viet Nam" OR Vietnam OR Cambodia OR Laos OR "Lao PDR" OR Myanmar OR Singapore OR Brunei) → 1543<br>3. ("antibiotic resistance" OR "antimicrobial resistance" OR "multidrug resistant" OR "MDR" OR "ESBL" OR "carbapenem resistant" OR "MRSA" OR "VRE" OR "drug resistant bacteria") AND ("antibiotic stewardship" OR "antimicrobial stewardship" OR "ASP" OR "infection prevention" OR "infection control" OR surveillance OR intervention* OR program* OR polic* OR guidelines) AND (effectiveness OR evaluation OR impact OR outcome*) AND (ASEAN OR "Southeast Asia" OR "South-East Asia" OR Indonesia OR Malaysia OR Thailand OR Vietnam OR "Viet Nam" OR Cambodia OR Laos OR Myanmar OR Singapore OR Brunei) → 416<br>4. ("antimicrobial resistance" OR "antibiotic resistance" OR "drug resistant infection*" OR "AMR") AND ("public health intervention*" OR "health system intervention*" OR "national action plan" OR "AMR surveillance" OR "One Health" OR "behavior change" OR "community intervention" OR "awareness campaign" OR "regulation" OR "policy") AND (effectiveness OR evaluation OR implement* OR impact) AND (ASEAN OR "Southeast Asia" OR "South-East Asia" OR Indonesia OR Malaysia OR Thailand OR Vietnam OR "Viet Nam" OR Cambodia OR Laos OR Myanmar OR Singapore OR Brunei) → 143<br>**TOTAL BEFORE DUPLICATE : 3278**<br>**TOTAL AFTER DUPLICATE : 2014**

**Cleaning**	Remove Duplicates	**Pubmed : 391**<br>**Science : 2219**<br>**Scopus : 2014**<br>**Total : 4624**<br>**TOTAL REMOVE DUPLICATES ALL: 5208**
	Based on Title and Abstract	**114**
	Based on Availability	**89**
	Based on Eligibility	**57**
	Included studies	**57**

PRISMA

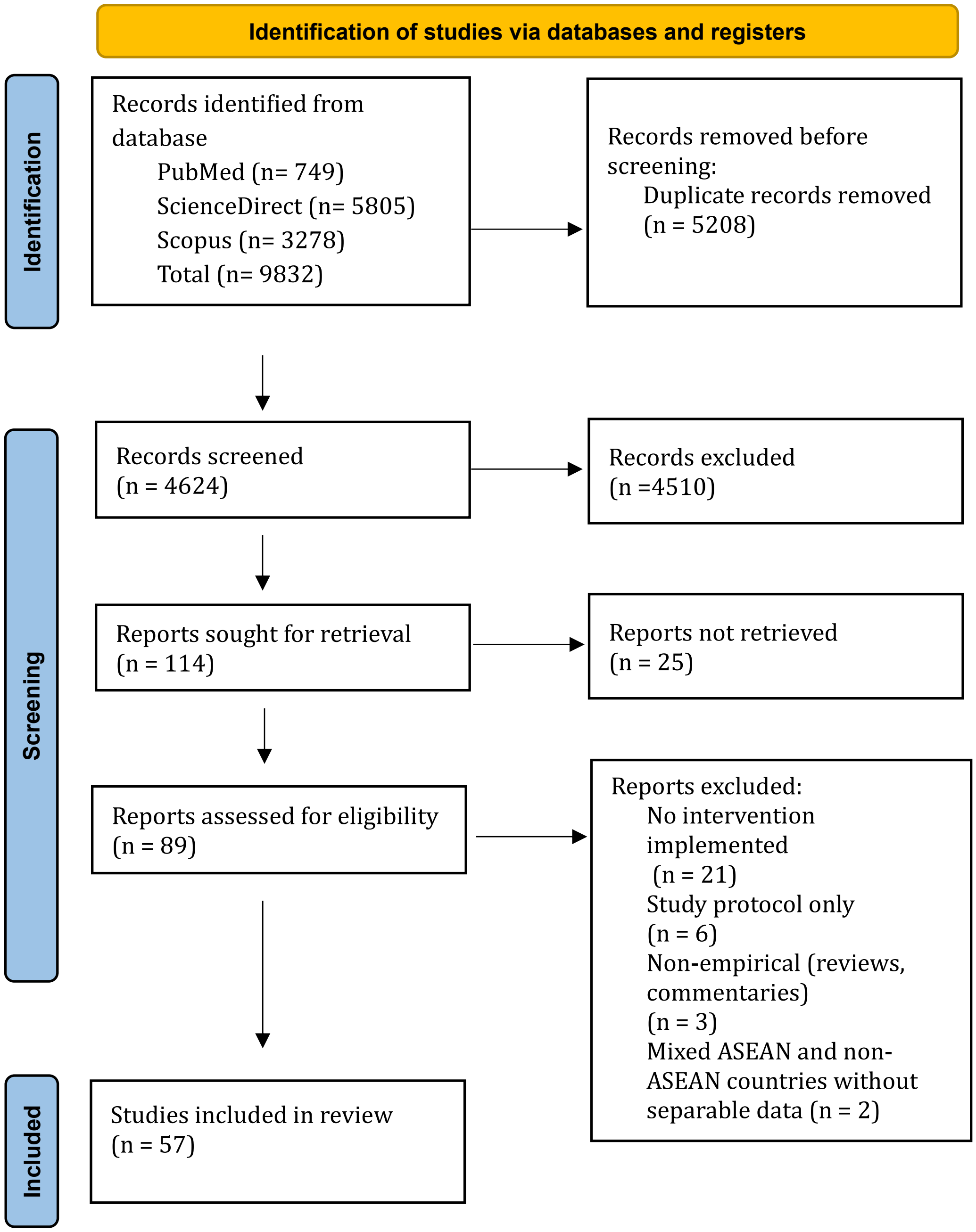
Identification of studies via databases and registers
Identification
Records identified from database
PubMed (n= 749)
ScienceDirect (n= 5805)
Scopus (n= 3278)
Total (n= 9832)
Records removed before screening:
Duplicate records removed (n = 5208)
Screening
Records screened (n = 4624)
Records excluded (n =4510)
Reports sought for retrieval (n = 114)
Reports not retrieved (n = 25)
Reports assessed for eligibility (n = 89)
Reports excluded:
No intervention implemented (n = 21)
Study protocol only (n = 6)
Non-empirical (reviews, commentaries) (n = 3)
Mixed ASEAN and non-ASEAN countries without separable data (n = 2)
Included
Studies included in review (n = 57)

Part C: Data Extraction Form

The following variables were systematically extracted from each included study:

1. **Study identification:** First author, publication year, title
2. **Country and setting:** Country, sector (human/animal/environmental/multisectoral), setting type
3. **Intervention characteristics:** Category, description
4. **Intervention activity:** Specific activities or components implemented
5. **One Health framework:** Pillar(s) addressed (intervention/surveillance/policy and economics/behavioral insights/multi-pillar)
6. **Study design:** Observational, quasi-experimental, experimental, mixed-methods, qualitative, or other
7. **Outcomes:** Primary outcome(s) measured
8. **Key findings:** Summary of main results and direction of effect

The form was piloted on five randomly selected studies and refined before full extraction commenced. Data extraction was independently performed by two reviewers (R.N. and S.A.Q.), with disagreements resolved through discussion or consultation with a third reviewer (M.J.).
